# Unilateral biportal endoscopic transforaminal lumbar interbody fusion versus minimally invasive transforaminal lumbar interbody fusion for single-level lumbar spondylolisthesis: a systematic review and meta-analysis

**DOI:** 10.3389/fmed.2025.1686492

**Published:** 2025-11-24

**Authors:** Yu Zhang, Jidong Ju, Jinchun Wu

**Affiliations:** Department of Orthopaedics, Jingjiang People’s Hospital Affiliated to Yangzhou University, Jingjiang, Jiangsu, China

**Keywords:** ULIF, MIS-TLIF, lumbar spondylolisthesis, efficacy, meta-analysis

## Abstract

**Objective:**

As an emerging surgical technique, the potential advantages of unilateral biportal endoscopic transforaminal lumbar interbody fusion (ULIF) for lumbar spondylolisthesis have yet to be substantiated by robust evidence. This study aims to investigate effectiveness and security of ULIF compared to minimally invasive transforaminal lumbar interbody fusion (MIS-TLIF) in managing single-level lumbar spondylolisthesis.

**Methods:**

We conducted a comprehensive search in six databases for publications comparing ULIF with MIS-TLIF for lumbar spondylolisthesis, systematically reviewing literature up until July 19, 2025. Meta-analyses were carried out via Stata 17.0 software.

**Results:**

Twelve studies met our inclusion criteria. Compared with MIS-TLIF, ULIF demonstrated significantly reduced intraoperative blood loss [WMD = −35.71, 95% CI (−51.80, −19.63), *p* < 0.01], fewer intraoperative fluoroscopy times [WMD = −1.29, 95% CI (−2.56, −0.02), *p* < 0.05], lower postoperative drainage volume [WMD = −20.64, 95% CI (−37.13, −4.15), *p* = 0.01], shorter postoperative ambulation time [WMD = −0.30, 95% CI (−0.42, −0.17), *p* < 0.01], and decreased hospital stay duration [WMD = −1.50, 95% CI (−2.09, −0.90), *p* < 0.01]. Additionally, patients undergoing ULIF exhibited improved visual analog scale scores for back pain [WMD = −0.09, 95% CI (−0.16, −0.02), *p* = 0.01] and leg pain [WMD = −0.09, 95% CI (−0.16, −0.03), *p* = 0.01] and Oswestry disability index [WMD = −0.77, 95% CI (−1.21, −0.32), *p* < 0.01] at final follow-up. Conversely, surgical duration for MIS-TLIF was significantly shorter than that for ULIF [WMD = 18.63, 95% CI (9.39, 27.87), *p* < 0.01]. No significant differences were observed between both groups regarding disc height, lumbar lordosis, fusion rates, or complication rates (*p* > 0.05).

**Conclusion:**

In comparison to MIS-TLIF, ULIF presents several advantages including less intraoperative blood loss, reduced reliance on fluoroscopy, diminished postoperative drainage, earlier ambulation capabilities after surgery, shorter hospital stay as well as enhanced recovery from back and leg pain along with improved lumbar function in patients affected by lumbar spondylolisthesis. However, ULIF requires more operative time than MIS-TLIF.

**Systematic review registration:**

https://www.crd.york.ac.uk/PROSPERO/view/CRD42025111069, CRD420251110694.

## Introduction

With aging population, lumbar spondylolisthesis (LS) has emerged as a progressively prevalent condition among the elderly demographic. It is a disorder characterized by the displacement of vertebrae, which compromises the stability of the intervertebral disc and leads to compression of adjacent neural and vascular structures within spinal canals (1, 2). LS typically results in persistent lower back pain that usually radiates to legs, accompanied by additional discomfort including numbness, weakness, and restricted movements (3). LS not only threatens patients’ quality of life but also results in significant economic and medical burdens for society. Epidemiological studies reveal LS influences around 5% among general populations, with a notably higher prevalence among older individuals (4). Surgical intervention is generally recommended for patients experiencing uncontrolled pain despite conservative treatment options (5, 6). It is reported that surgical management of lumbar spondylolisthesis is superior to nonsurgical approaches (7).

Transforaminal lumbar interbody fusion (TLIF) was initially introduced as a classical procedure in 1982 (8). Since then, it has gained recognition as an effective strategy for managing lumbar spondylolisthesis. TLIF involves facet joint resections, spinal canal decompressions, and interbody fusions executed via unilateral intervertebral approach. Primary objectives of TLIF are threefold: to restore disc height, relieve nerve compression, and enhance spinal stabilization. However, conventional posteromedial approach during TLIF entails bilateral dislocation and traction of multifidus muscle, which can result in a number of potential adverse outcomes, including inflammatory response, muscular injury, tissue scarring, and denervation of the paravertebral muscle tissue (9). Such consequences may directly diminish spinal flexion strength, which has the potential to result in postoperative low back pain and subsequent complication (10).

To reduce healthcare-related injuries and postoperative complications associated with traditional TLIF, there has been a trend toward the adoption of minimally invasive TLIF (MIS-TLIF) (11, 12). MIS-TLIF integrates a conventional open decompression with minimally invasive approach, leading to less invasive fusion process (13). However, clinical practice has revealed that MIS-TLIF is characterized by an insufficient working channel and restricted operational area (14). Several studies have indicated an escalation in the frequency of revision surgeries, readmissions, and hardware-related complications, as well as an augmented occurrence of nerve root injuries associated with MIS-TLIF (15, 16).

As the popularity of minimally invasive techniques continues to grow, propelled by rapid advancements in endoscopic methods, the development of endoscopic approaches for spinal surgery has accelerated significantly (17). Unilateral biportal endoscopic TLIF (ULIF) has garnered increasing favor among spinal surgeons due to its unique advantages. Unlike traditional open surgical procedures, ULIF utilizes both one working portal and one observation portal, which together provide a wider operative field and enhanced flexibility, contributing to improved operational efficiency. ULIF minimizes damage to surrounding paravertebral muscles and ligamentous structures while preserving segmental stability. ULIF is frequently employed for treatment of spinal stenosis and disc herniation and may achieve effective decompression (18, 19).

ULIF and MIS-TLIF represent significant advancements within the minimally invasive spectrum of TLIF techniques. Previous meta-analyses have compared ULIF with MIS-TLIF in lumbar degenerative diseases (20–24). While ULIF generally demonstrates superiority over MIS-TLIF, the pooled results remain contentious. This controversy may arise from the inclusion of various conditions involving disc herniation, spinal stenosis, and spondylolisthesis in these analyses, which could introduce bias into pooled results. Despite considerable interest from spine surgeons regarding the potential benefits of ULIF, there is a notable lack of meta-analysis specifically comparing ULIF with MIS-TLIF for lumbar spondylolisthesis. Our study aims to fill this knowledge gap by performing the first meta-analysis to evaluate the effectiveness and security of ULIF versus MIS-TLIF specifically for single-level lumbar spondylolisthesis. The findings from this study are anticipated to exert a substantial influence on clinical practice and play a significant role in the development of future clinical guidelines.

## Methods

This study was conducted in accordance with the Preferred Reporting Items for Systematic Reviews and Meta-Analyses (PRISMA) guidelines (25).

### Search strategy

Literature search was performed in the following databases: PubMed, Embase, Web of Science, Cochrane Library, China National Knowledge Infrastructure, and Wanfang Database from inception to July 19, 2025. Languages were limited to Chinese and English. A detailed description of search strategy and formula is provided in Supplementary file 1.

### Eligibility

#### Inclusion criteria

1. Population: Patients diagnosed with single-level lumbar spondylolisthesis.
2. Intervention: ULIF.
3. Comparison: MIS-TLIF.
4. Outcomes: Operation time, intraoperative blood loss, intraoperative fluoroscopy times, postoperative drainage volume, postoperative ambulation time, hospital stay, visual analog scale (VAS) score for back and leg pain, Oswestry disability index (ODI), disc height, lumbar lordosis, fusion rate, and complication rates.
5. Study design: Randomized controlled trials (RCTs) or observational studies.
6. Follow-up duration: At least 1 year.

#### Exclusion criteria

1. Animal experiments, reviews, case reports, conference abstracts, or meta-analyses.
2. Studies with insufficient information for data extraction.
3. Publications with unclear or erroneous data.
4. Duplicate publications.
5. Articles not published in English or Chinese.

### Data extraction

Data were extracted using standardized forms, including:

1. Study characteristics: first author, publication year, country, and study design.
2. Patient information: sample size, age, follow-up duration, and Meyerding grade.
3. Predefined outcomes.

### Quality assessment

Quality of RCTs was appraised via Cochrane Risk of Bias Tool (26). With regard to observational studies, Newcastle-Ottawa Scale was utilized to assess study quality (27).

### Statistical analysis

Meta-analysis was conducted using Stata 17.0. For dichotomous outcomes, odds ratios (ORs) and 95% confidence intervals (CIs) were calculated. For continuous outcomes, weighted mean differences (WMDs) and 95% CIs were employed. I^2^ statistics was used to assess heterogeneity across included investigations. I^2^ > 50% was considered significant heterogeneity, in which case a random-effects model was applied for pooling. Otherwise, a fixed-effects model was utilized. When considerable heterogeneity was observed, meta-regression and subgroup analyses were conducted to explore potential sources of heterogeneity. Potential contributors to heterogeneity included publication year, country, and Meyerding grade. Sensitivity analyses were performed to evaluate the robustness of pooled results by sequentially excluding individual studies and recalculating overall effect. Publication bias was assessed using Egger test. *p* < 0.05 was considered statistically significant.

## Results

### Search process

**Figure 1:**
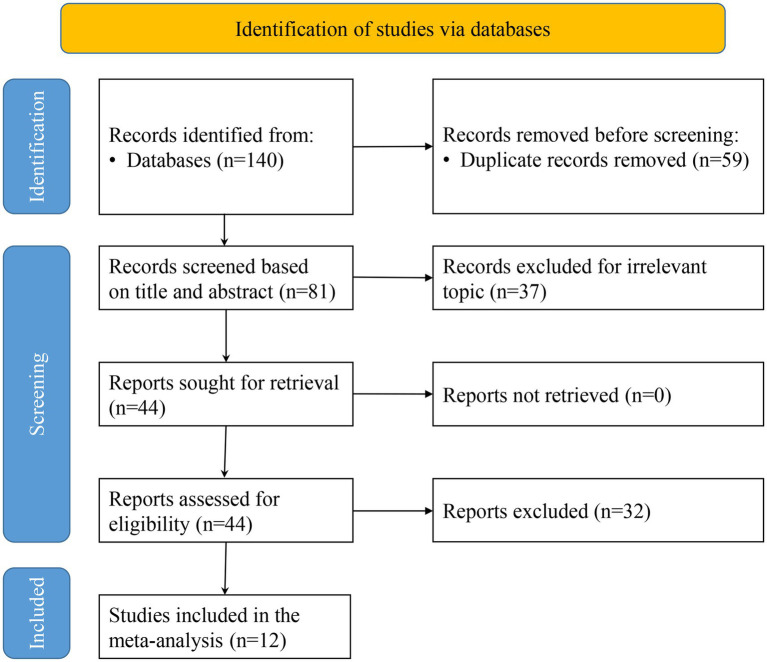
Flow chart of literature search.

An initial comprehensive search of online databases yielded 140 potentially relevant articles. After removing 59 duplicates, 81 articles remained for title and abstract screening, resulting in the exclusion of 37 studies that did not meet the inclusion criteria. Full-text evaluation of the remaining 44 articles led to the final inclusion of 12 studies (28–39) (Figure 1).

### Study characteristics

Of the eligible papers, 11 were retrospective studies and 1 was a RCT, involving 1,038 patients, with 515 assigned to ULIF group and 523 to MIS-TLIF group. Ten researches were carried out in China, one in Indonesia, and one in South Korea. All studies were published between 2021 and 2025 (Table 1).

**Table 1:** Study characteristics.

Author	Year	Country	Study design	Meyerding grade	Sample size		Age (years)		Follow-up time (months)	
					ULIF	MIS-TLIF	ULIF	MIS-TLIF	ULIF	MIS-TLIF
Zhu	2025	China	RS	I	28	27	64.2 ± 5.4	65.7 ± 4.8	14.1 ± 1.4	14.7 ± 1.6
Lu	2024	China	RS	I	26	28	59 ± 14.4	61.7 ± 13.3	14.3 ± 3.2	12.9 ± 2.0
Kim	2021	Korea	RS	I	32	55	70.5 ± 8.26	67.3 ± 10.7	27.2 ± 5.4	31.5 ± 7.3
Song	2022	China	RS	I	28	28	54.7 ± 10.0	56.3 ± 11.6	14.1 ± 1.5	14.3 ± 1.4
He	2025	China	RS	I-II	28	21	59.5 ± 7.6	60.2 ± 6.6	16.4 ± 3.9	17.6 ± 3.2
Bahir	2024	China	RS	I-II	40	45	64.77 ± 5.68	65.64 ± 5.21	24	24
Guo	2023	China	RS	I-II	26	23	64.15 ± 6.42	66.09 ± 6.10	24	24
Gatam	2021	Indonesia	RS	I-II	72	73	55.1 ± 5.12	52.3 ± 6.13	12	12
Feng	2024	China	RS	I-II	50	56	57.4 ± 5.8	58.2 ± 6.0	16.5 ± 3.1	16.5 ± 3.1
Pan	2024	China	RS	I-II	30	32	55.1 ± 12.5	56.0 ± 13.7	12 ~ 18	12 ~ 18
Yu	2023	China	RS	I-II	23	18	60.8 (45 ~ 74)	60.7 (46 ~ 71)	38.96 ± 3.17	39.50 ± 3.35
Sang	2024	China	RCT	II-III	132	117	60.3 ± 8.6	60.7 ± 9.1	15.0 ± 3.0	15.0 ± 3.0

ULIF, unilateral biportal endoscopic transforaminal lumbar interbody fusion; MIS-TLIF, minimally invasive transforaminal lumbar interbody fusion; RS, retrospective study; RCT, randomized controlled trial.

### Quality assessment

As for observational studies, quality assessment is detailed in Table 2. Of the included observational studies, 3 were rated as 7 points and 8 as 8 points, indicating high quality.

**Table 2:** Newcastle-Ottawa Scale of observational studies.

Study	Selection	Comparability	Outcome	Total score
Zhu et al. (39)	3	1	3	7
He et al. (38)	4	1	3	8
Lu et al. (35)	4	1	3	8
Bahir et al. (33)	4	1	3	8
Guo et al. (31)	4	1	3	8
Gatam et al. (28)	4	1	3	8
Kim et al. (29)	4	1	3	8
Feng et al. (34)	3	1	3	7
Pan et al. (36)	4	1	3	8
Yu et al. (32)	4	1	3	8
Song et al. (30)	3	1	3	7

**Figure 2:**
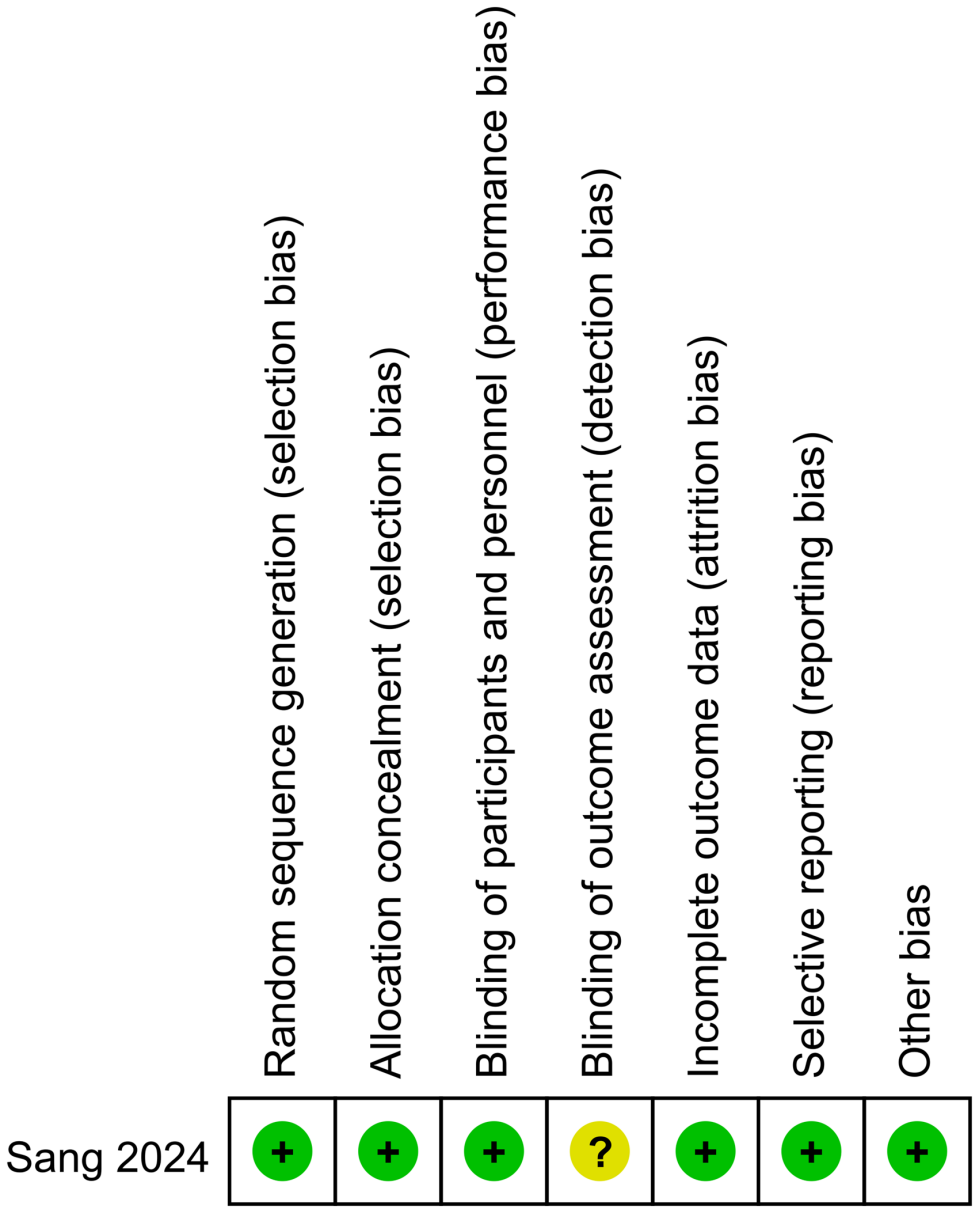
Cochrane risk of bias assessment.

Figure 2 reflects the risk of bias in RCT. The result suggested that detection bias was not clear and the overall risk of bias was not high.

### Meta-analysis results

Table 3 presents pooled results of 13 predefined outcomes.

**Table 3:** Findings of meta-analysis.

Outcome	Study size	Effect size	95% CI		*p*-value	Heterogeneity	Effect model	Egger test
		WMD/OR	Lower limit	Upper limit		I^2^ (%)		*p*-value
Operative time	11	18.63	9.39	27.87	<0.01	91.94	Random	0.87
Blood loss	9	−35.71	−51.80	−19.63	<0.01	96.96	Random	0.40
Fluoroscopy times	2	−1.29	−2.56	−0.02	<0.05	87.68	Random	1.00
Drainage volume	3	−20.64	−37.13	−4.15	0.01	90.31	Random	0.89
Ambulation time	3	−0.30	−0.42	−0.17	<0.01	58.40	Random	0.11
Hospital day	10	−1.50	−2.09	−0.90	<0.01	77.64	Random	0.95
VAS (back pain)	9	−0.09	−0.16	−0.02	0.01	0.00	Fixed	0.67
VAS (leg pain)	9	−0.09	−0.16	−0.03	0.01	0.00	Fixed	0.69
ODI	9	−0.77	−1.21	−0.32	<0.01	0.00	Fixed	0.34
Disc height	8	0.01	−0.26	0.28	0.94	50.92	Random	0.87
Lumbar lordosis	10	−0.05	−0.47	0.37	0.82	10.66	Fixed	0.27
Fusion	10	1.02	0.61	1.72	0.93	0.00	Fixed	0.54
Complication	11	0.72	0.44	1.20	0.21	0.00	Fixed	0.20

WMD, weighted mean diference; OR, odds ratio; CI, confidence interval; VAS, visual analog scale; ODI, Oswestry disability index.

#### Operative time

**Figure 3:**
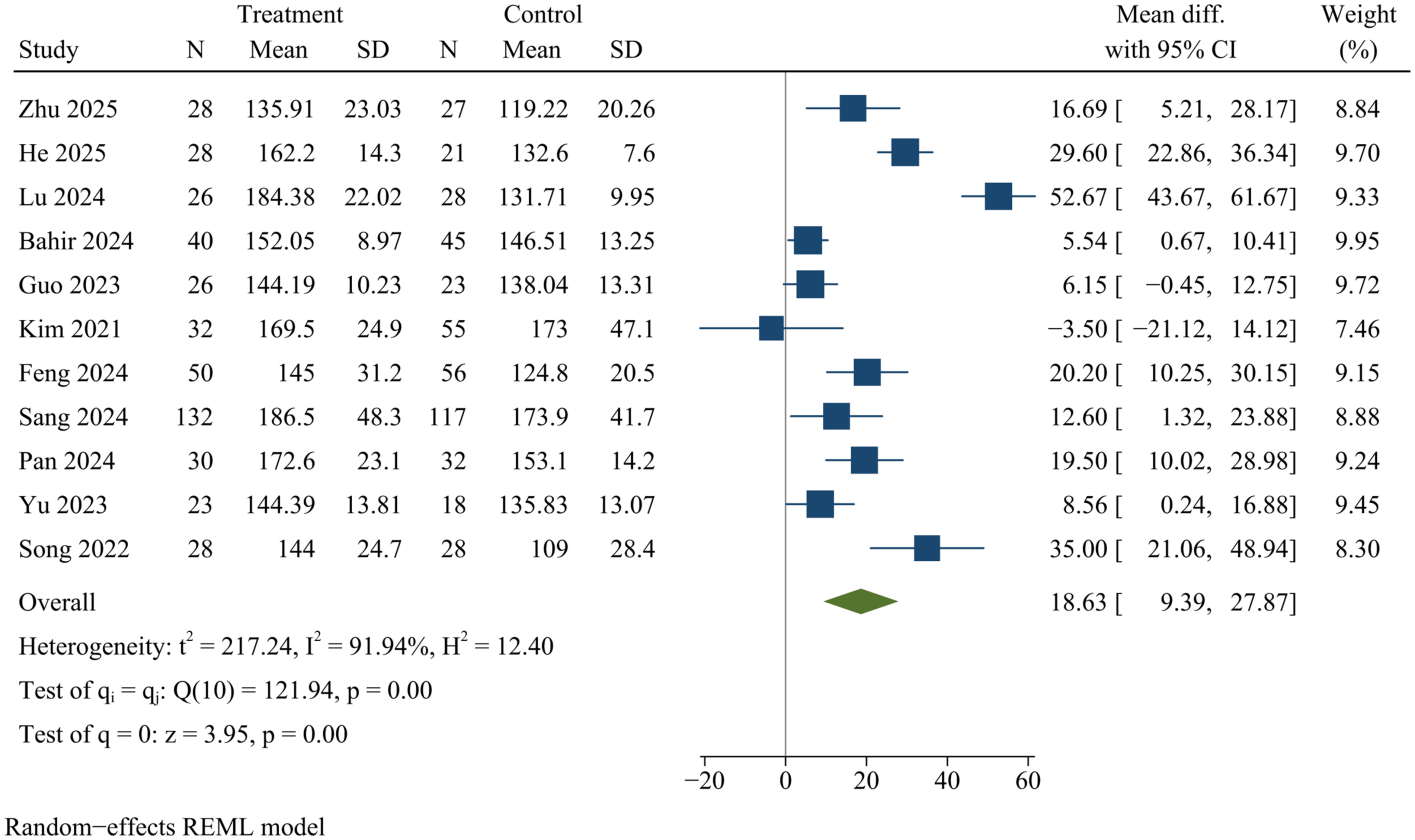
Forest plot of surgical duration.

Eleven studies reported operative time. ULIF was linked to a significantly prolonged surgical duration compared with MIS-TLIF [WMD = 18.63, 95% CI (9.39, 27.87), *p* < 0.01] (Figure 3).

#### Intraoperative bleeding

**Figure 4:**
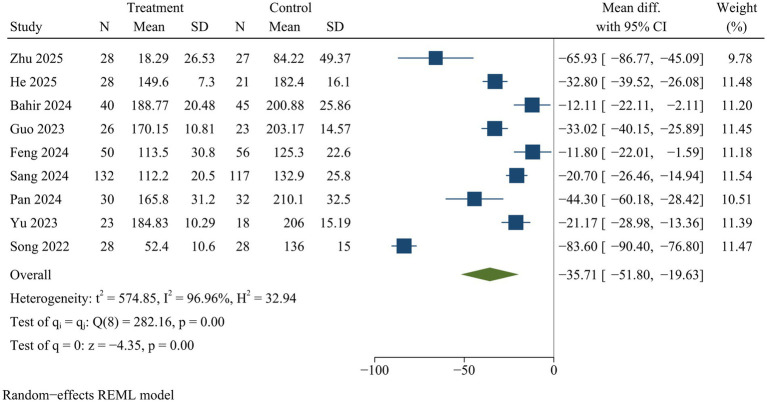
Forest plot of intraoperative bleeding.

Nine studies reported intraoperative blood loss. ULIF significantly reduced intraoperative blood loss compared with MIS-TLIF [WMD = −35.71, 95% CI (−51.80, −19.63), *p* < 0.01] (Figure 4).

#### Intraoperative fluoroscopy times

**Figure 5:**
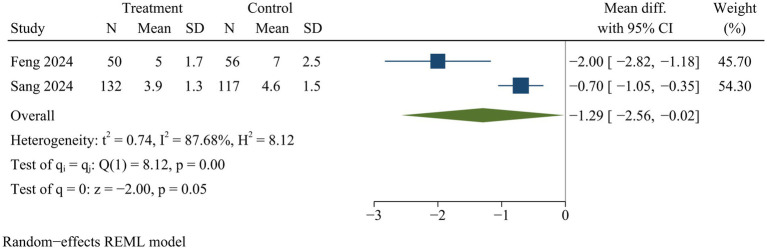
Forest plot of fluoroscopy times.

Two studies provided data on intraoperative fluoroscopy times. ULIF significantly reduced fluoroscopic times during surgery compared with MIS-TLIF [WMD = −1.29, 95% CI (−2.56, −0.02), *p* < 0.05] (Figure 5).

#### Postoperative drainage

**Figure 6:**
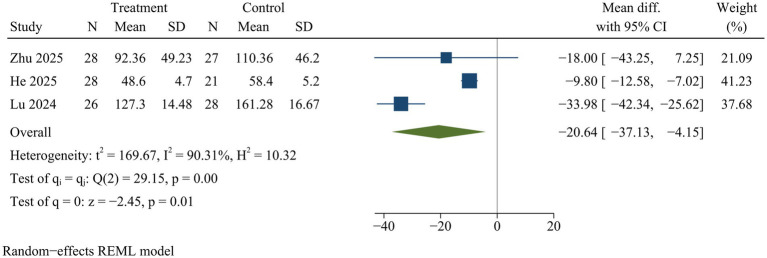
Forest plot of drainage volume.

Three studies reported postoperative drainage volume. ULIF significantly reduced postoperative drainage volume compared with MIS-TLIF [WMD = −20.64, 95% CI (−37.13, −4.15), *p* = 0.01] (Figure 6).

#### Postoperative ambulation time

**Figure 7:**
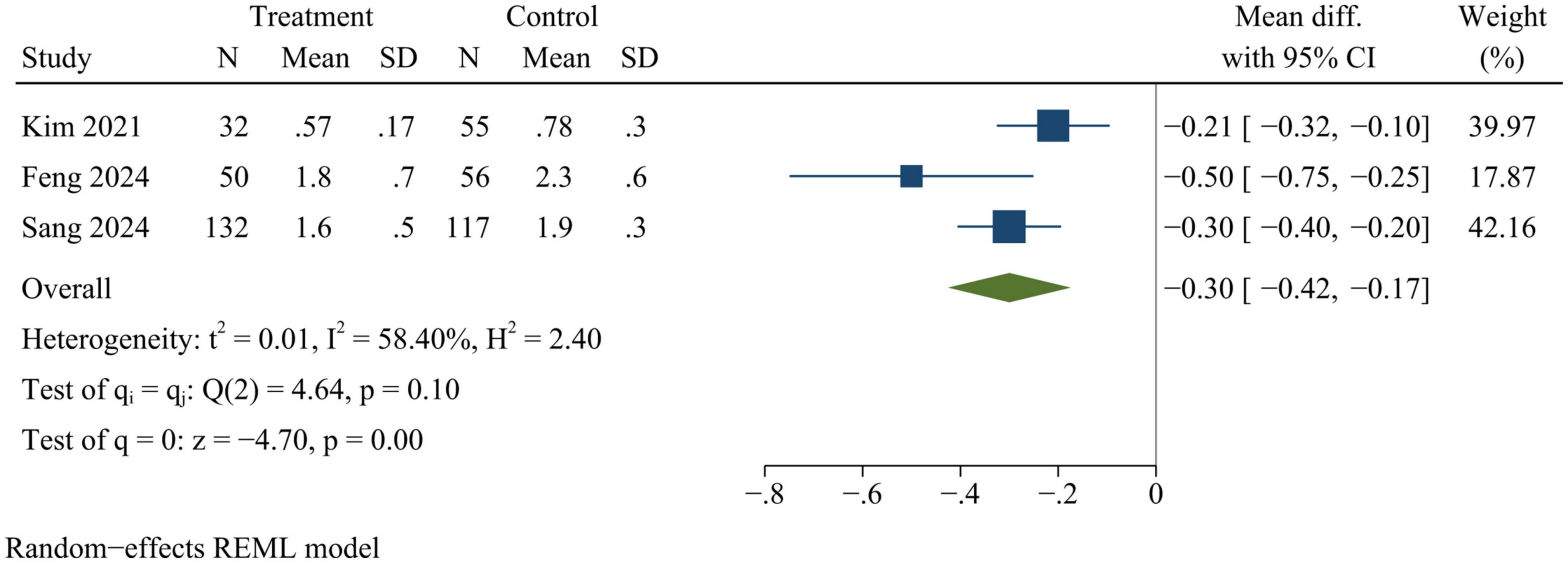
Forest plot of ambulation time.

Three studies evaluated ambulation time after surgery. ULIF significantly shortened ambulation time after surgery compared with MIS-TLIF [WMD = −0.30, 95% CI (−0.42, −0.17), *p* < 0.01] (Figure 7).

#### Hospital stay

**Figure 8:**
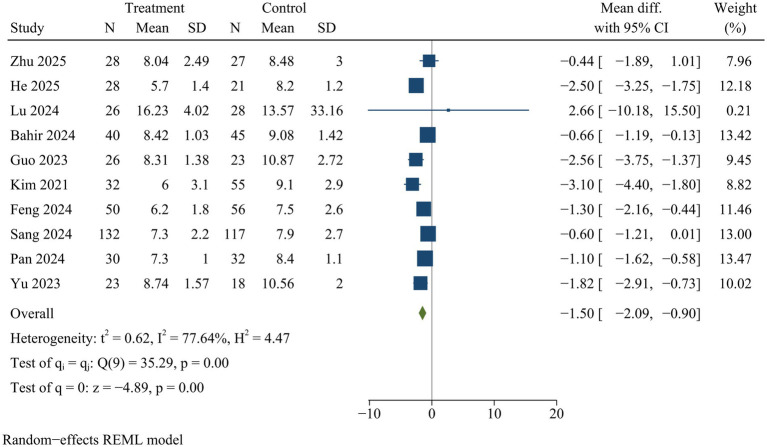
Forest plot of hospital day.

Ten studies provided data on hospitalization duration. ULIF significantly reduced the length of hospital stay compared with MIS-TLIF [WMD = −1.50, 95% CI (−2.09, −0.90), *p* < 0.01] (Figure 8).

#### VAS

**Figure 9:**
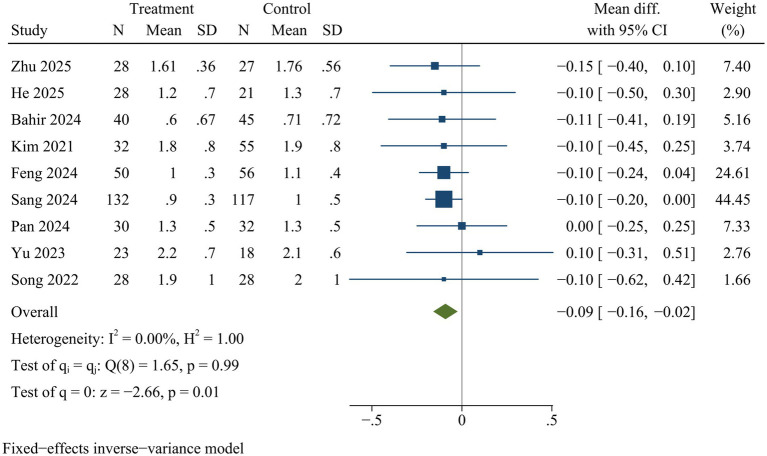
Forest plot of visual analog scale (back pain).

**Figure 10:**
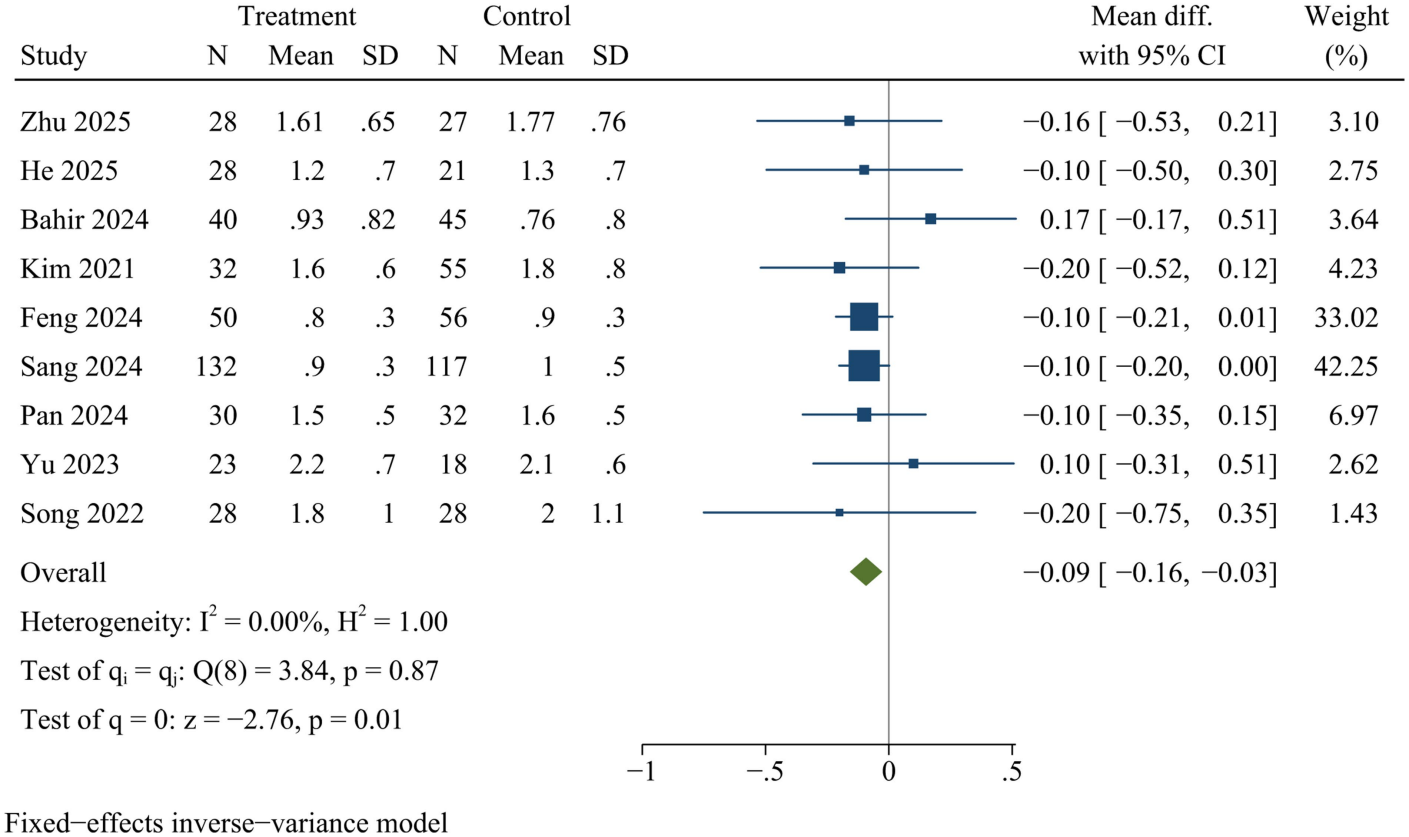
Forest plot of visual analog scale (leg pain).

Nine studies reported VAS. VAS scores for back pain [WMD = −0.09, 95% CI (−0.16, −0.02), *p* = 0.01] (Figure 9) and leg pain [WMD = −0.09, 95% CI (−0.16, −0.03), *p* = 0.01] (Figure 10) were significantly lower in ULIF group compared with MIS-TLIF group at final follow-up.

#### ODI

**Figure 11:**
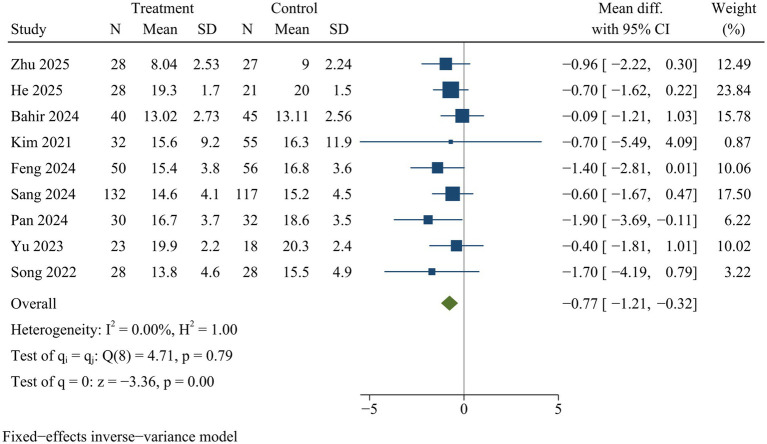
Forest plot of Oswestry disability index.

Nine studies reported ODI. ULIF significantly improved functional outcomes, as indicated by lower ODI, compared with MIS-TLIF at final follow-up [WMD = −0.77, 95% CI (−1.21, −0.32), *p* < 0.01] (Figure 11).

#### Disc height

**Figure 12:**
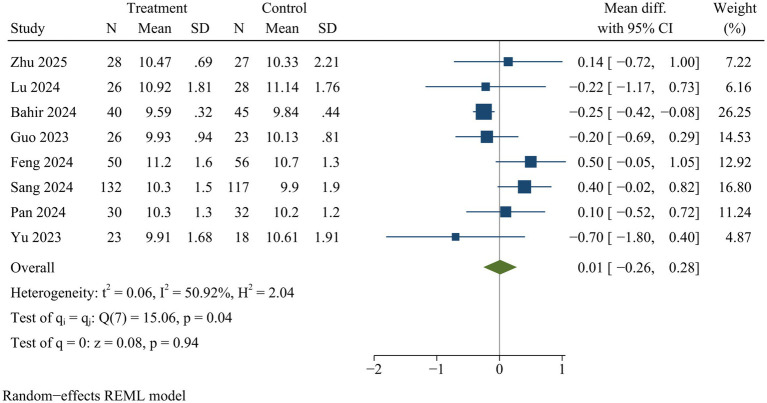
Forest plot of disc height.

Eight studies measured disc height at final follow-up. No statistically significant difference in disc height was observed between ULIF and MIS-TLIF groups [WMD = 0.01, 95% CI (−0.26, 0.28), *p* = 0.94] (Figure 12).

#### Lumbar lordosis

**Figure 13:**
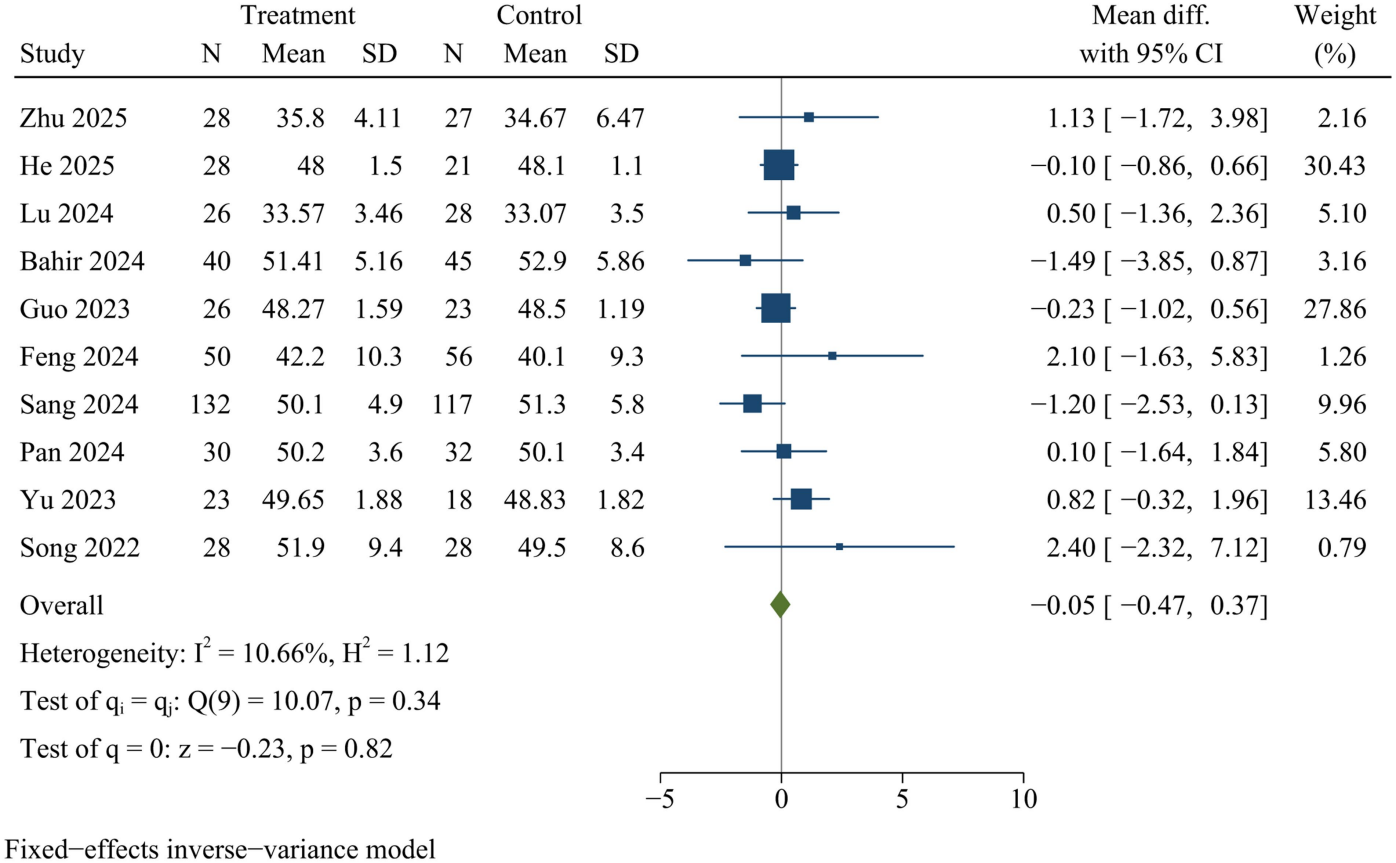
Forest plot of lumbar lordosis.

Ten studies assessed lumbar lordosis at final follow-up. There was no significant difference in lumbar lordosis between both surgical approaches [WMD = −0.05, 95% CI (−0.47, 0.37), *p* = 0.82] (Figure 13).

#### Fusion

**Figure 14:**
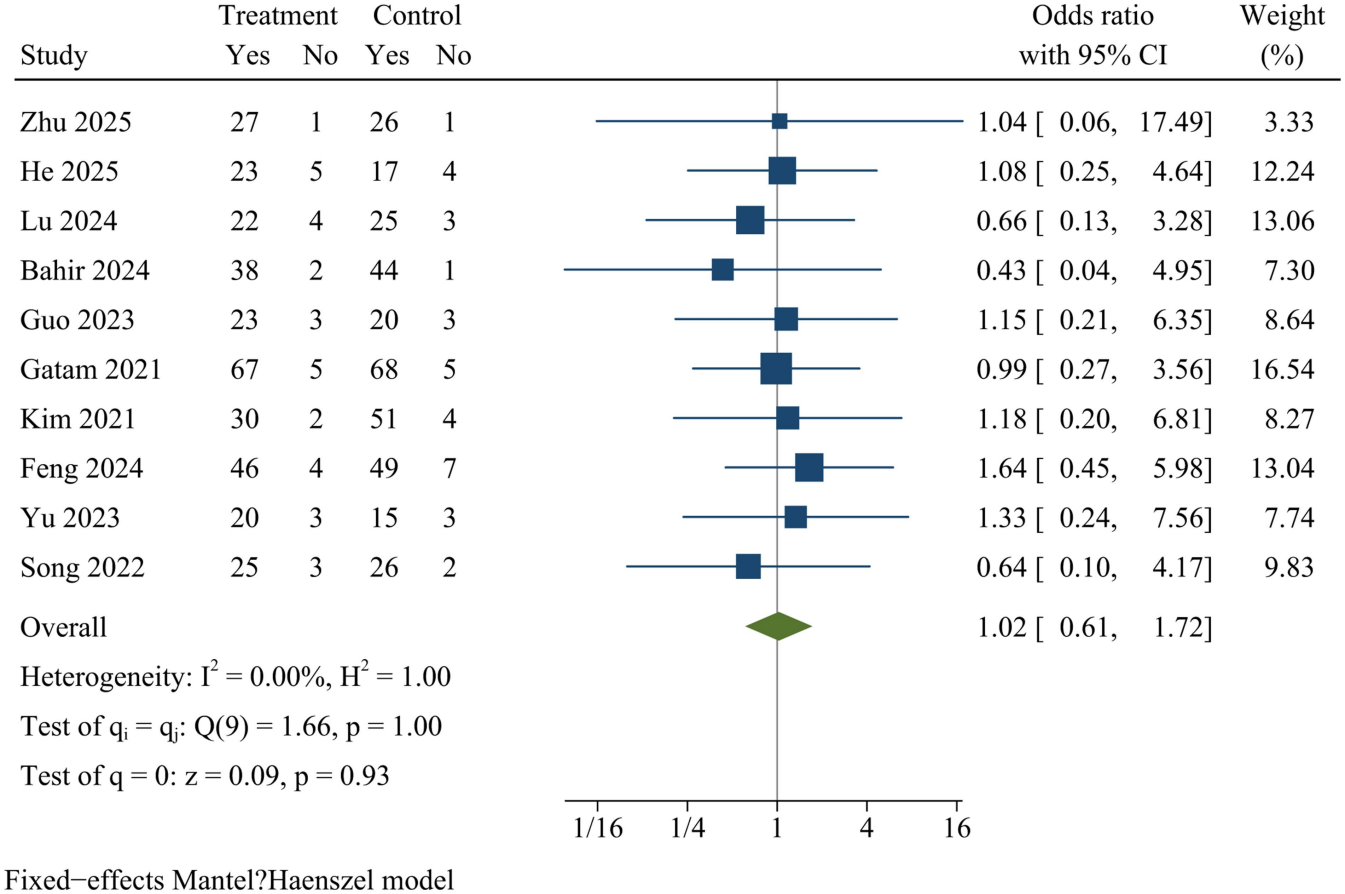
Forest plot of fusion.

Ten studies compared fusion rates. The pooled analysis showed no significant difference in fusion rates between ULIF and MIS-TLIF at final follow-up [OR = 1.02, 95% CI (0.61, 1.72), *p* = 0.93] (Figure 14).

#### Complication

**Figure 15:**
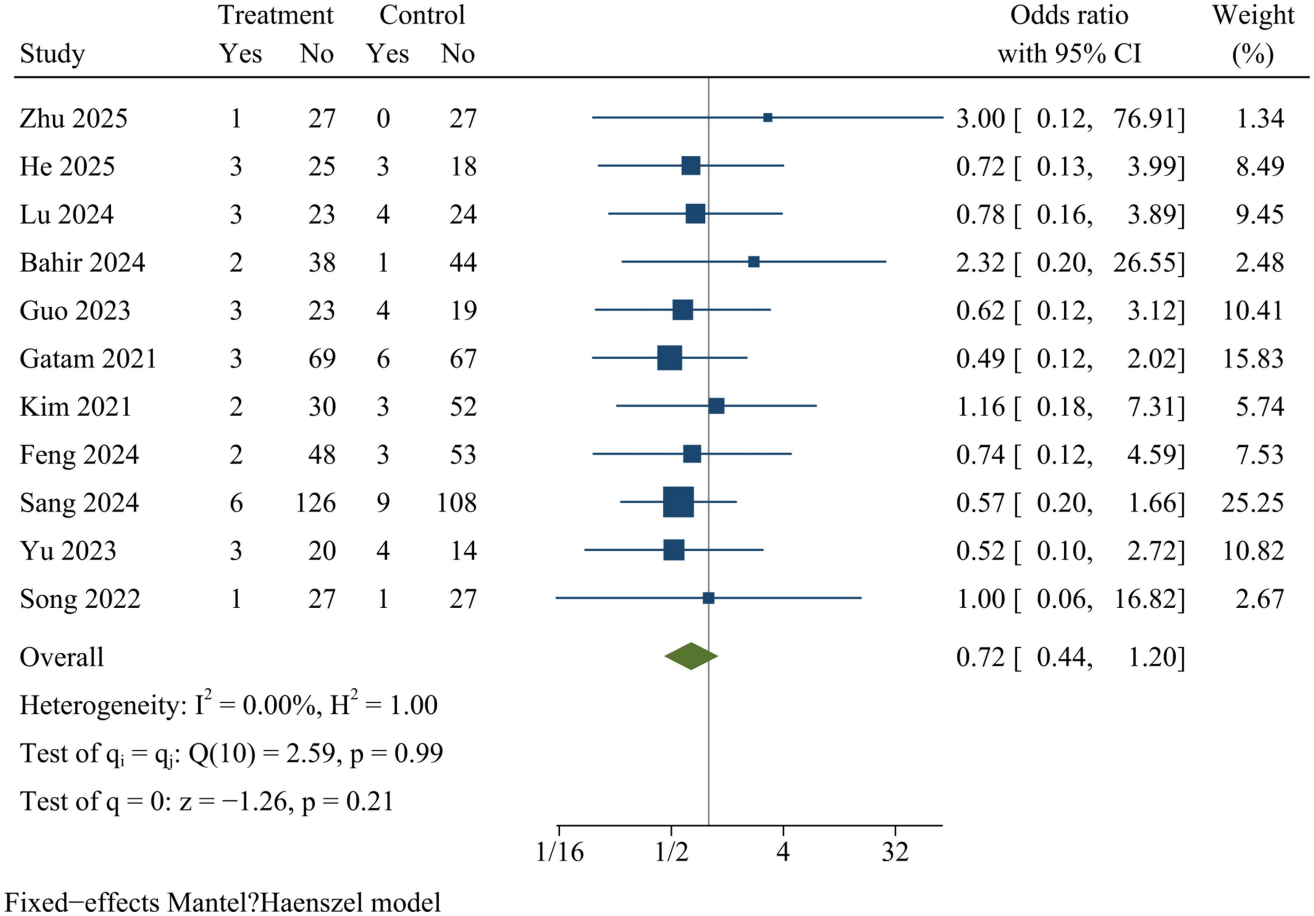
Forest plot of complications.

Eleven studies documented postoperative complications. The meta-analysis revealed no significant difference in complication rates between ULIF and MIS-TLIF groups [OR = 0.72, 95% CI (0.44, 1.20), *p* = 0.21] (Figure 15).

### Meta-regression analysis for potential heterogeneity sources

Significant heterogeneity was observed across studies for operative duration, intraoperative bleeding, fluoroscopy times, drainage volume, ambulation time, hospital stay, and disc height. Univariate meta-regression analyses were conducted to explore potential sources of heterogeneity. Due to limited studies, analyses for intraoperative fluoroscopy times, postoperative drainage volume, and ambulation time were not feasible. The results indicated neither publication year, country, nor Meyerding grade significantly contributed to heterogeneity. Therefore, no further subgroup analyses were performed (Supplementary file 2).

### Sensitivity analysis

Sensitivity analysis indicated there were no statistically significant changes in the effect sizes of outcome indicators after excluding individual trials, with the exception of drainage volume and back VAS scores. Consequently, the findings related to drainage volume and back VAS scores might not be stable, while pooled results for the remaining outcome measures were deemed reliable (Supplementary file 3).

### Publication bias

Egger test revealed no significant publication bias (Supplementary file 4).

## Discussion

### Background

Lumbar spondylolisthesis is a common condition encountered in clinical practice, particularly among elderly female patients. TLIF is widely regarded as the gold standard regarding spinal fusion surgeries. Nevertheless, conventional open procedures often result in substantial damage to spinal posterior region, which can negatively impact lumbar stability and frequently lead to postoperative complications (40). The advent of ULIF and MIS-TLIF represents notable advancements in minimally invasive surgical techniques within this domain. Nevertheless, there has been a lack of meta-analyses specifically comparing ULIF and MIS-TLIF for lumbar spondylolisthesis. Our study aims to fill this gap by presenting the first meta-analysis that compares ULIF with MIS-TLIF for single-level lumbar spondylolisthesis. The insights gained from this research are anticipated to significantly influence clinical practice and contribute meaningfully to future clinical guidelines.

### Main findings

Our findings indicated that ULIF significantly led to reduced intraoperative bleeding, fewer intraoperative fluoroscopy times, decreased postoperative drainage volume, less ambulation duration, shorter hospitalization duration, lower VAS scores for both back and leg pain, as well as improved ODI at final follow-up when compared with MIS-TLIF for managing single-level lumbar spondylolisthesis. Nevertheless, while ULIF demonstrated these advantages, MIS-TLIF significantly exhibited a shorter operative duration than ULIF. At final follow-up, no significant differences were observed between ULIF and MIS-TLIF concerning disc height, lumbar lordosis, fusion rate or complication incidence. Therefore, our findings suggest that ULIF significantly reduces surgical trauma and intraoperative fluoroscopy exposure while effectively alleviating pain and promoting postoperative functional recovery compared to MIS-TLIF for lumbar spondylolisthesis.

### Meta-analysis result discussion

#### Operative time

Our meta-analysis indicated that ULIF was linked to a considerably extended operative duration in comparison to MIS-TLIF. The extended operative duration can be attributed to the relatively recent development of ULIF and its ongoing maturation process. For novice surgeons, there may also be instances of misidentification of surgical segments, which further contributes to an increased procedural length. The inherent requirements of ULIF necessitate unique manual coordination; one hand must secure the endoscopes while maintaining an unobstructed visual field, whereas the other hand performs intricate maneuvers within a confined surgical workspace. Additionally, it is essential to maintain an uninterrupted intraoperative fluid catheter, complicating matters as the endoscope is employed for complex procedures such as laminectomy, discectomy, and endplate preparation. The intricacy involved in these procedures, coupled with inherent limitations, reasonably accounts for the prolonged duration characteristic of ULIF. Moreover, the lengthy learning curve associated with ULIF may also elucidate why procedure duration is extended when surgeons are not yet familiar with anatomically marked structures through endoscopic visualization. Research has demonstrated that surgical technique tends to stabilize after approximately 34 cases (41). During the early stages on this learning curve, surgeries typically require more time due to insufficient experience. However, once surgeons attain adequate proficiency, operation duration decreases significantly. Therefore, it is strongly recommended that spine surgeons achieve a high level of competence in ULIF and attain a steady state on their learning curve prior to performing ULIF procedure. This ensures that patients are not adversely affected by the extended duration required for surgery. One contributing factor to prolonged surgical duration lies in the occurrence of bleeding from small vessels and bone surfaces intraoperatively, which can compromise visibility in the surgical field. Therefore, meticulous intraoperative hemostasis utilizing a radiofrequency electrotome to maintain a lucid operative view is of paramount importance. This underscores the imperative for systematic instruction and continuing education within the trauma and spine community (28).

#### Intraoperative fluoroscopy times

Our analysis revealed that MIS-TLIF significantly increased intraoperative fluoroscopy times compared to ULIF. Feng and Sang et al. suggested that MIS-TLIF presented specific challenges, including restricted visual field, constrained operating space, and compromised surgical visibility due to bleeding, which consequently became essential to enhance fluoroscopy times to facilitate MIS-TLIF procedure (34, 37). We found that several previous meta-analyses did not compare intraoperative fluoroscopy times, probably due to insufficient data (20, 22, 24). Our study included data from only two studies, and there was significant heterogeneity. Therefore, the interpretation of the findings regarding fluoroscopy times needs to be cautious. Significant heterogeneity may be caused by experience and learning curve of surgeons.

#### Bleeding and drainage

The findings of this study indicated that ULIF significantly reduced both intraoperative bleeding and postoperative drainage compared with MIS-TLIF. However, caution should be exercised when interpreting these estimates regarding blood loss accuracy. Given that ULIF employs water-based technology, accurately quantifying the extent of bleeding poses considerable challenges. The observed lower levels of blood loss and postoperative drainage in ULIF may stem from saline irrigation fluid perfusion utilized throughout ULIF operative decompression as well as intervertebral bone grafting, which generates specific water pressure that aids in achieving pressurized hemostasis. The combination of continuous saline irrigation alongside radiofrequency electrocoagulation effectively minimizes bleeding while enhancing surgical visibility (42).

#### Ambulation time and hospital stay

The results from this study demonstrated that ULIF significantly shortened both postoperative ambulation time and hospital stay in comparison to MIS-TLIF. ULIF involves less soft tissue dissection, and unremitting saline irrigation facilitates a reduction in the generation of inflammatory factors, thereby protecting soft tissues posterior to spine through ULIF. Early mobilization and resumption of functional exercises postoperatively are beneficial for patients’ recovery. Furthermore, unlike MIS-TLIF, ULIF obviates the need for the insertion of tubular retractors into the posterior paraspinal muscles, thereby minimizing the occurrence of direct ischemic injury to muscles. Although MIS-TLIF offers a unique operative strategy, extensive dissection of the posterior spinal soft tissue may prolong recovery time, delay ambulation, and extend hospital stays (20). Lower levels of C-reactive protein have been observed in the ULIF group, indicating that ULIF may reduce soft tissue damage and inflammatory responses (43, 44).

#### VAS and ODI

The reduction in back and leg pain following spinal surgeries is crucial for assessing surgical techniques. Our results demonstrated VAS scores for back pain and leg pain, and ODI were significantly less in ULIF compared to MIS-TLIF at final follow-up. Our findings indicate ULIF may effectively alleviate low back pain and facilitate functional recovery. Such results are consistent with previous studies (45). When effective neural decompression is achieved, the severity of paraspinal muscular retraction and dissection becomes a pivotal factor associated with subsequent lower back discomfort. Compared to the distractor utilized in MIS-TLIF, tension-free endoscopy markedly shortens the period of paraspinal muscular contraction. It also minimizes risks associated with muscular damage, denervation, and ischemic trauma, which are associated with postoperative low back syndrome, resulting in lower VAS scores for back pain at final follow-up. Consequently, ULIF provides greater relief from postoperative back pain and improves postoperative functional recovery compared to MIS-TLIF.

The efficacy of neural decompression is related to the extent to which leg pain is alleviated following lumbar surgeries. The neural decompression capability of ULIF is highly regarded. Although direct decompression during MIS-TLIF is fairly simplistic, it faces limitations due to restricted access and visualization. In contrast, ULIF allows for more precise decompression through continuous irrigation and enhanced visualization afforded by its biportal system, especially in complex cases (43). Our findings indicated that ULIF significantly reduced leg pain VAS scores compared to MIS-TLIF, suggesting that ULIF might provide superior nerve decompression relative to MIS-TLIF.

#### Negative indicators

Radiological outcomes serve as primary indicators for evaluating lumbar fusion techniques, taking into account factors such as disc height restoration, lordosis angle correction, and fusion rate. Nevertheless, no significant differences were observed regarding fusion rates. The fusion rates are crucial metrics for assessing surgical efficacy. Continuous saline irrigation during ULIF, particularly within fusion bed in intervertebral area, may potentially diminish blood supply and osteogenic factors, which could adversely affect fusion. Nevertheless, no significant difference regarding lumbar fusion rate was identified between both groups at final follow-up. This finding indicates both ULIF and MIS-TLIF achieve satisfactory fusion rates. This finding aligns with results from previous studies (46). Furthermore, proper preparation of endplate implantation beds is crucial for successful spinal fusions, and endplate bleeding serves as an indicator of appropriate bed preparation. However, traditional interbody fusion techniques, including MIS-TLIF, depend on manual manipulation for endplate preparation, which can result in incomplete removal of cartilage or damage to the bony endplate. In contrast, ULIF facilitates direct visualization and precise manipulation of the endplate, thereby creating a favorable environment for subsequent implant placement and enhanced chances of successful fusions. Additional findings revealed that, at the final follow-up, there were no significant differences in lumbar lordosis or disc height between both groups. These results suggest ULIF and MIS-TLIF are both effective in restoring normal spinal alignment and ensuring postsurgical lumbar stability.

Common complications associated with ULIF involve dural tears, cerebrospinal fluid leakage, spinal epidural hematoma, nerve root injuries and so on. Among these, dural tear or cerebrospinal fluid leakage are considered the most significant complications within ULIF (47). While ULIF enhances intraoperative visualization, it may inadvertently exert traction on the dura mater, which may lead to dural tear and subsequent cerebrospinal fluid leakage postoperatively. This risk is particularly pronounced in cases where surgeons have limited experience with endoscopic systems. Factors such as a steep learning curve, intraoperative bleeding events, or suboptimal surgical visualization due to low saline irrigation pressure or obstructed outflow can further exacerbate this issue. Consequently, effective management of intraoperative bleeding and enhancement of surgical stability are paramount. This study found no statistically significant differences regarding surgical complications between ULIF and MIS-TLIF. This finding suggests that the safety of these two surgical approaches are comparable.

#### Heterogeneity analysis

This study identified substantial heterogeneity across various studies concerning operative duration, intraoperative bleeding, intraoperative fluoroscopy times, drainage volumes, ambulation time, length of hospital stay, and disc height. Due to limited available researches, meta-regression analyses were not conducted for intraoperative fluoroscopy times, postoperative drainage volume, and ambulation time. Potential sources of heterogeneity were presumed to include variations in publication year, country, and Meyerding grade. Univariate meta-regression analysis was conducted to investigate these potential sources. However, the results indicated that publication year, country, and Meyerding grade did not significantly contribute to heterogeneity. The considerable heterogeneity in length of hospital stay might be attributed to differences in healthcare insurance systems as well as individual physiological variations. Additionally, factors such as the type and duration of non-surgical treatments, including medications, physical therapies, and targeted nerve blocks, might also play a role. The observed heterogeneity in intraoperative blood loss could stem from discrepancies in surgical techniques employed for hemostasis and variations in methods used for quantifying blood loss across different studies. Furthermore, the variability in operative time might be caused by the learning curve related to the surgeons’ clinical experience.

### Strengths

To the best of our knowledge, this is the first meta-analysis specifically comparing ULIF and MIS-TLIF for single-level lumbar spondylolisthesis. This systematic review possesses several strengths that enhance its validity. First, literature search was comprehensively undertaken encompassing six major online databases, ensuring extensive coverage of relevant studies. Second, stringent inclusion and exclusion criteria were applied to minimize potential confounding factors and bolster the reliability of the findings. Third, Egger test was performed to assess publication bias, revealing no significant publication bias. Fourth, univariate meta-regression analyses were undertaken to explore potential sources of heterogeneity. Consequently, this study holds significant clinical relevance and warrants careful interpretation as it provides robust evidence supporting the use of ULIF in treating lumbar spondylolisthesis. Additionally, these findings may inform the design of future large-scale, high-quality RCTs.

### Limitations

Despite these strengths, this meta-analysis had several limitations. Firstly, most studies were retrospective in nature, which restricted the ability to control for clinical heterogeneity and selection bias. Secondly, nearly all of the existing investigations were carried out in China, potentially introducing biases related to local healthcare practices, clinical protocols, and patient characteristics that might affect the generalizability of our findings. Thirdly, significant heterogeneity was observed in multiple outcomes. Although univariate meta-regression analyses were performed, sources of heterogeneity could not be clearly identified, impacting the evidence’s reliability. Consequently, the pooled results might need to be interpreted with caution. Fourthly, follow-up duration across studies were generally short, thus limiting our ability to assess long-term outcomes effectively. Fifthly, only studies in Chinese or English were included, consequently increasing the risk of language bias and potentially omitting relevant literature from other languages. Finally, comprehensive comparison between both procedures was impeded by a lack of data regarding medical costs.

### Implications for future research

Our findings indicate ULIF may present advantages over MIS-TLIF in lumbar spondylolisthesis. However, further RCTs are necessary to validate these results and to conduct a thorough evaluation of the benefits and limitations associated with each technique. Additionally, large-scale, multicenter trails can strengthen the evidence base by enhancing sample size and patient diversity. International collaborative efforts can further substantiate these findings and improve their generalizability. Future research should prioritize this area by including more studies in order to facilitate a comprehensive assessment of adverse events. Furthermore, a critical objective for subsequent investigations is the comparative cost-effectiveness analysis between ULIF and MIS-TLIF. Such analyses would provide healthcare policymakers with vital data regarding the economic implications of adopting ULIF. We advocate for the development of comparative clinical studies assessing ULIF versus MIS-TLIF across various grades of lumbar spondylolisthesis. These studies would elucidate its therapeutic efficacy and refine indications for its application based on spondylolisthesis severity. Lastly, it is essential to investigate the surgical learning curve associated with ULIF adoption. Subsequent studies should assess the impact of surgeon experience and training on patients’ prognoses to ensure the secure and successful adoption of ULIF.

## Conclusion

This study demonstrated that ULIF was superior to MIS-TLIF in reducing intraoperative bleeding, fluoroscopy times, postoperative drainage, ambulation duration, hospital stay, and VAS and ODI scores at final follow-up for single-level lumbar spondylolisthesis. However, MIS-TLIF significantly shortened operation time. Future research should include well-designed RCTs and further studies to explore the efficacy and security of ULIF in optimizing long-term prognosis and quality of life for patients with lumbar spondylolisthesis.

## Data Availability

The original contributions presented in the study are included in the article/Supplementary material, further inquiries can be directed to the corresponding author.
