# Supplementary material for: Unilateral biportal endoscopic transforaminal lumbar interbody fusion versus minimally invasive transforaminal lumbar interbody fusion for single-level lumbar spondylolisthesis: a systematic review and meta-analysis
Source: Front Med (Lausanne). 2025 Nov 24;12:1686492. doi: 10.3389/fmed.2025.1686492 (PMC12682878; doi:10.3389/fmed.2025.1686492)
Supplement: Supplementary file 1 [file Data_Sheet_1.pdf]

The search formula is outlined as follows:
((((((((unilateral biportal endoscopy) OR (unilateral biportal endoscopic)) OR (biportal endoscopic)) OR (biportal endoscopic spinal surgery)) OR (irrigation endoscopic discectomy)) OR (two portal endoscopic spinal surgery)) OR (twoportal endoscopic spinal surgery)) AND (((minimally invasive transforaminal lumbar interbody fusion) OR (MIS-TLIF)) OR (MI-TLIF))) AND (lumbar spondylolisthesis)

This systematic review and meta-analysis adhered to the Preferred Reporting Items for Systematic Reviews and Meta-analyses (PRISMA) guidelines, ensuring transparency and comprehensive reporting. Furthermore, this study has been registered with the International Registry of Prospective Systematic Reviews (PROSPERO). Ethical approval and patient consent were not required for this research, as all analyses were based on previously published studies.

Two researchers independently conducted the literature search, study selection, data extraction, and quality assessment. They subsequently cross-verified their results to ensure consistency and resolve any discrepancies. In cases of disagreement between the two investigators during any phase of the process, discussions were held until a consensus was reached. If consensus could not be achieved, the corresponding author was consulted to make a final determination.

EndNote 20 software was utilized to organize literature from multiple databases. Based on predefined inclusion criteria, two researchers screened the literature independently. After eliminating duplicate studies, irrelevant articles were excluded through a review of titles and abstracts. Ultimately, full texts were examined to identify eligible studies. To prevent omissions, reference lists from relevant articles and reviews were manually scrutinized for additional studies. For studies involving overlapping populations, data were extracted from those with the largest sample size; in instances where sample sizes matched across studies, data extraction prioritized the most recently published work. Data extraction from included studies was performed using standardized tables.
