# Supplementary material for: Unilateral biportal endoscopic transforaminal lumbar interbody fusion versus minimally invasive transforaminal lumbar interbody fusion for single-level lumbar spondylolisthesis: a systematic review and meta-analysis
Source: Front Med (Lausanne). 2025 Nov 24;12:1686492. doi: 10.3389/fmed.2025.1686492 (PMC12682878; doi:10.3389/fmed.2025.1686492)
Supplement: Supplementary file 2 [file Data_Sheet_2.pdf]

Meta-regression analysis of

Duration of Surgery

```
. meta regress year

  Effect-size label: Mean diff.
        Effect size: _meta_es
          Std. err.: _meta_se

Random-effects meta-regression                    Number of obs  =        11
Method: REML                                      Residual heterogeneity:
                                                              tau2 =   216.5
                                                            I2 (%) =   91.93
                                                                H2 =   12.40
                                                     R-squared (%) =    0.35
                                                  Wald chi2(1)   =      1.03
                                                  Prob > chi2    =    0.3097

------------------------------------------------------------------------------
     _meta_es | Coefficient  Std. err.      z    P>|z|     [95% conf. interval]
--------------+----------------------------------------------------------------
         year |   4.324378   4.256471     1.02   0.310    -4.018151    12.66691
        _cons |  -8732.201   8613.413    -1.01   0.311    -25614.18    8149.778
------------------------------------------------------------------------------
Test of residual homogeneity: Q_res = chi2(9) = 107.72   Prob > Q_res = 0.0000

. meta regress country

  Effect-size label: Mean diff.
        Effect size: _meta_es
          Std. err.: _meta_se

Random-effects meta-regression                    Number of obs  =        11
Method: REML                                      Residual heterogeneity:
                                                              tau2 =   201.6
                                                            I2 (%) =   91.97
                                                                H2 =   12.45
                                                     R-squared (%) =    7.21
                                                  Wald chi2(1)   =      1.87
                                                  Prob > chi2    =    0.1712

------------------------------------------------------------------------------
     _meta_es | Coefficient  Std. err.      z    P>|z|     [95% conf. interval]
--------------+----------------------------------------------------------------
      country |  -23.89359   17.46031    -1.37   0.171    -58.11517    10.32799
        _cons |   44.28717   19.29254     2.30   0.022     6.474484    82.09987
------------------------------------------------------------------------------
Test of residual homogeneity: Q_res = chi2(9) = 116.91   Prob > Q_res = 0.0000

. meta regress meyerding_grade

  Effect-size label: Mean diff.
        Effect size: _meta_es
          Std. err.: _meta_se

Random-effects meta-regression                    Number of obs  =        11
Method: REML                                      Residual heterogeneity:
                                                              tau2 =     201
                                                            I2 (%) =   91.60
                                                                H2 =   11.91
                                                     R-squared (%) =    7.46
                                                  Wald chi2(1)   =      1.41
                                                  Prob > chi2    =    0.2353

--------------------------------------------------------------------------------
       _meta_es | Coefficient  Std. err.      z    P>|z|     [95% conf. interval]
----------------+----------------------------------------------------------------
meyerding_gr~e |  -.8142541   .6861324    -1.19   0.235    -2.159049    .5305407
          _cons |   26.17562   7.818205     3.35   0.001     10.85222    41.49902
--------------------------------------------------------------------------------
Test of residual homogeneity: Q_res = chi2(9) = 92.14    Prob > Q_res = 0.0000

.
```

Meta-regression analysis of

Hospital Stay

```
. meta regress year

  Effect-size label: Mean diff.
        Effect size: _meta_es
          Std. err.: _meta_se

Random-effects meta-regression                    Number of obs  =        10
Method: REML                                      Residual heterogeneity:
                                                              tau2 =   .5362
                                                            I2 (%) =   75.81
                                                                H2 =    4.13
                                                     R-squared (%) =   13.83
                                                  Wald chi2(1)   =      2.75
                                                  Prob > chi2    =    0.0970

    _meta_es | Coefficient  Std. err.      z    P>|z|     [95% conf. interval]
-------------+----------------------------------------------------------------
        year |   .4570959   .2754683     1.66   0.097    -.0828121    .9970039
       _cons |  -926.5367   557.4793    -1.66   0.097    -2019.176    166.1027
------------------------------------------------------------------------------
Test of residual homogeneity: Q_res = chi2(8) = 31.87    Prob > Q_res = 0.0001

. meta regress country

  Effect-size label: Mean diff.
        Effect size: _meta_es
          Std. err.: _meta_se

Random-effects meta-regression                    Number of obs  =        10
Method: REML                                      Residual heterogeneity:
                                                              tau2 =   .4579
                                                            I2 (%) =   73.17
                                                                H2 =    3.73
                                                     R-squared (%) =   26.41
                                                  Wald chi2(1)   =      3.20
                                                  Prob > chi2    =    0.0738

    _meta_es | Coefficient  Std. err.      z    P>|z|     [95% conf. interval]
-------------+----------------------------------------------------------------
     country |  -1.766784   .9882249    -1.79   0.074    -3.703669    .1701013
       _cons |   .4335674   1.104799     0.39   0.695    -1.731799    2.598934
------------------------------------------------------------------------------
Test of residual homogeneity: Q_res = chi2(8) = 27.17    Prob > Q_res = 0.0007

. meta regress meyerding_grade

  Effect-size label: Mean diff.
        Effect size: _meta_es
          Std. err.: _meta_se

Random-effects meta-regression                    Number of obs  =        10
Method: REML                                      Residual heterogeneity:
                                                              tau2 =   .5703
                                                            I2 (%) =   75.35
                                                                H2 =    4.06
                                                     R-squared (%) =    8.35
                                                  Wald chi2(1)   =      1.06
                                                  Prob > chi2    =    0.3023

      _meta_es | Coefficient  Std. err.      z    P>|z|     [95% conf. interval]
---------------+----------------------------------------------------------------
meyerding_gr~e |   .0509184   .0493579     1.03   0.302    -.0458214    .1476581
         _cons |  -2.082393   .6447854    -3.23   0.001    -3.346149   -.8186366
--------------------------------------------------------------------------------
Test of residual homogeneity: Q_res = chi2(8) = 28.88    Prob > Q_res = 0.0003

.
```

Meta-regression analysis of

Intraoperative Blood Loss

```
. meta regress year

  Effect-size label: Mean diff.
        Effect size: _meta_es
          Std. err.: _meta_se

Random-effects meta-regression                       Number of obs  =          9
Method: REML                                         Residual heterogeneity:
                                                                 tau2 =    584.7
                                                               I2 (%) =    96.82
                                                                   H2 =    31.49
                                                        R-squared (%) =     0.00
                                                     Wald chi2(1)    =      0.71
                                                     Prob > chi2     =    0.3996

------------------------------------------------------------------------------
    _meta_es | Coefficient  Std. err.      z    P>|z|     [95% conf. interval]
-------------+----------------------------------------------------------------
        year |   7.603449    9.02654     0.84   0.400    -10.08824    25.29514
       _cons |  -15423.24   18267.51    -0.84   0.399     -51226.9    20380.42
------------------------------------------------------------------------------
Test of residual homogeneity: Q_res = chi2(7) = 179.39   Prob > Q_res = 0.0000

. meta regress meyerding_grade

  Effect-size label: Mean diff.
        Effect size: _meta_es
          Std. err.: _meta_se

Random-effects meta-regression                       Number of obs  =          9
Method: REML                                         Residual heterogeneity:
                                                                 tau2 =      257
                                                               I2 (%) =    92.75
                                                                   H2 =    13.79
                                                        R-squared (%) =    55.29
                                                     Wald chi2(1)    =     10.10
                                                     Prob > chi2     =    0.0015

--------------------------------------------------------------------------------
      _meta_es | Coefficient  Std. err.      z    P>|z|     [95% conf. interval]
---------------+----------------------------------------------------------------
meyerding_gr~e |   2.895066   .9109568     3.18   0.001     1.109624    4.680509
         _cons |  -67.38451   11.55564    -5.83   0.000    -90.03316   -44.73587
--------------------------------------------------------------------------------
Test of residual homogeneity: Q_res = chi2(7) = 110.38   Prob > Q_res = 0.0000

.
```

Meta-regression analysis of

Disc Height

```
. meta regress year

  Effect-size label: Mean diff.
        Effect size: _meta_es
          Std. err.: _meta_se

Random-effects meta-regression                     Number of obs  =          8
Method: REML                                       Residual heterogeneity:
                                                               tau2 =  .06622
                                                             I2 (%) =   52.61
                                                                 H2 =    2.11
                                                      R-squared (%) =    0.00
                                                   Wald chi2(1)     =    1.19
                                                   Prob > chi2      =  0.2757

------------------------------------------------------------------------------
    _meta_es | Coefficient  Std. err.      z    P>|z|     [95% conf. interval]
-------------+----------------------------------------------------------------
        year |   .2977048   .2731027     1.09   0.276    -.2375667    .8329764
       _cons |  -602.5068   552.7266    -1.09   0.276    -1685.831    480.8175
------------------------------------------------------------------------------
Test of residual homogeneity: Q_res = chi2(6) = 14.18    Prob > Q_res = 0.0276

. meta regress meyerding_grade

  Effect-size label: Mean diff.
        Effect size: _meta_es
          Std. err.: _meta_se

Random-effects meta-regression                     Number of obs  =          8
Method: REML                                       Residual heterogeneity:
                                                               tau2 =  .05463
                                                             I2 (%) =   47.12
                                                                 H2 =    1.89
                                                      R-squared (%) =   13.32
                                                   Wald chi2(1)     =    0.98
                                                   Prob > chi2      =  0.3215

--------------------------------------------------------------------------------
      _meta_es | Coefficient  Std. err.      z    P>|z|     [95% conf. interval]
---------------+----------------------------------------------------------------
meyerding_gr~e |   .0216814   .0218702     0.99   0.322    -.0211833    .0645462
         _cons |   -.261314    .301948    -0.87   0.387    -.8531213    .3304932
--------------------------------------------------------------------------------
Test of residual homogeneity: Q_res = chi2(6) = 11.62    Prob > Q_res = 0.0710

.
```
