# Supplementary material for: Unilateral biportal endoscopic transforaminal lumbar interbody fusion versus minimally invasive transforaminal lumbar interbody fusion for single-level lumbar spondylolisthesis: a systematic review and meta-analysis
Source: Front Med (Lausanne). 2025 Nov 24;12:1686492. doi: 10.3389/fmed.2025.1686492 (PMC12682878; doi:10.3389/fmed.2025.1686492)

Sensitivity analysis of

Duration of Surgery

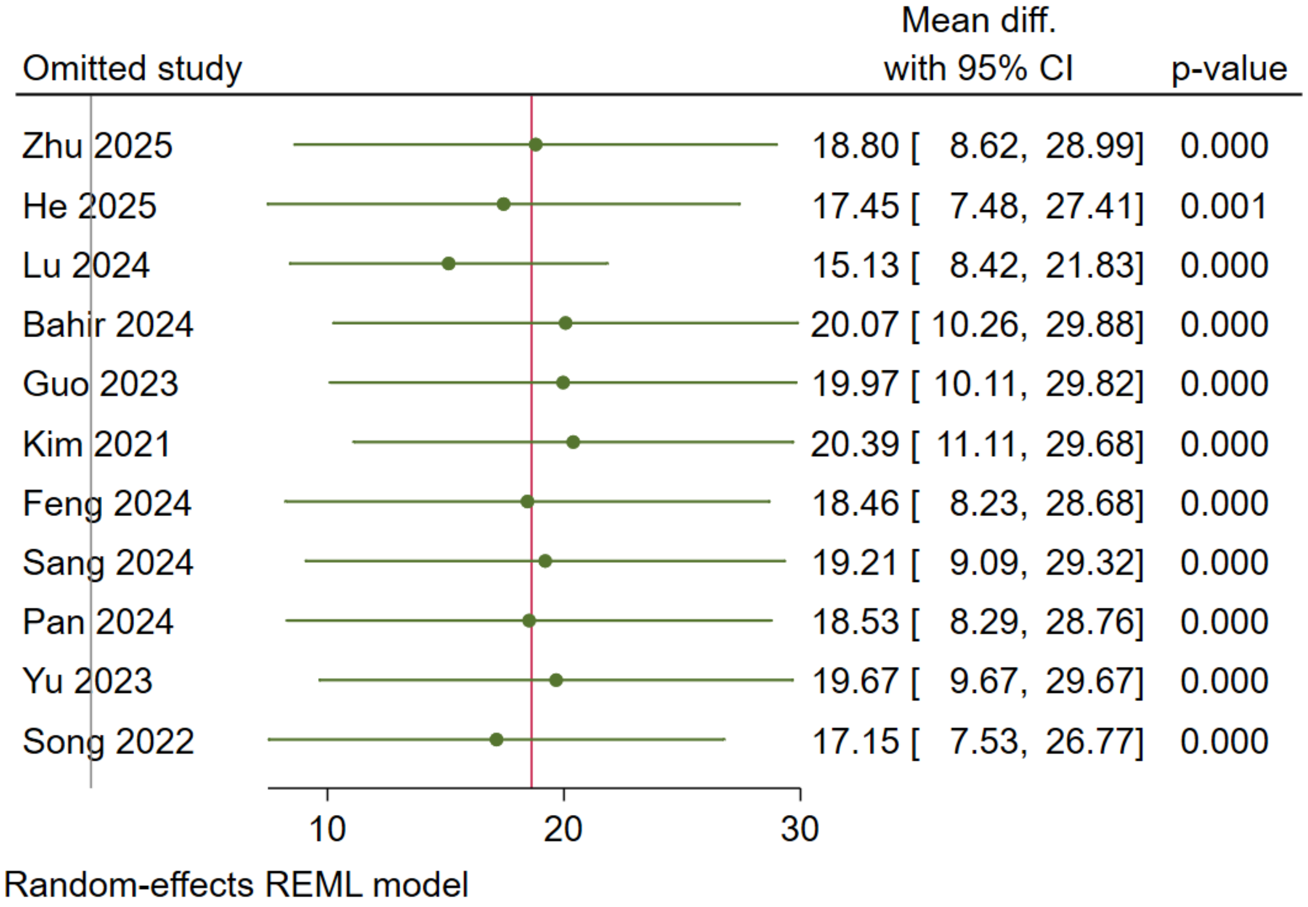

Sensitivity analysis of

Intraoperative Blood Loss

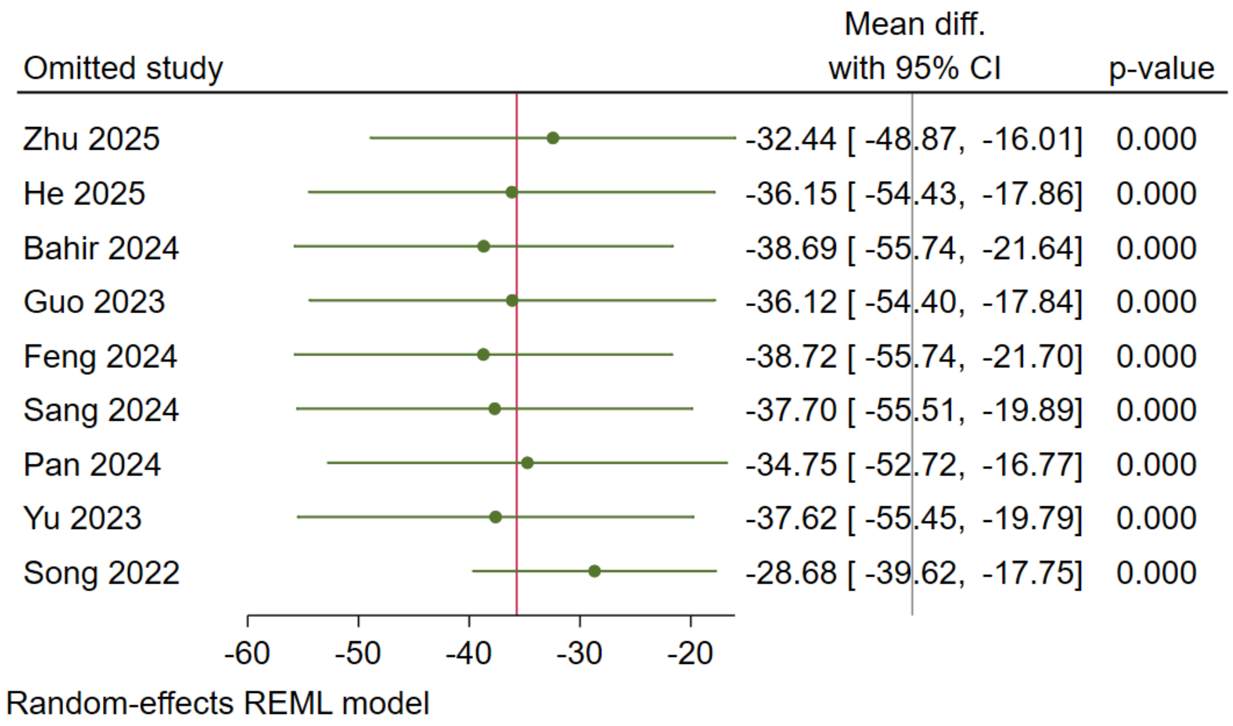

Sensitivity analysis of

Intraoperative Fluoroscopy

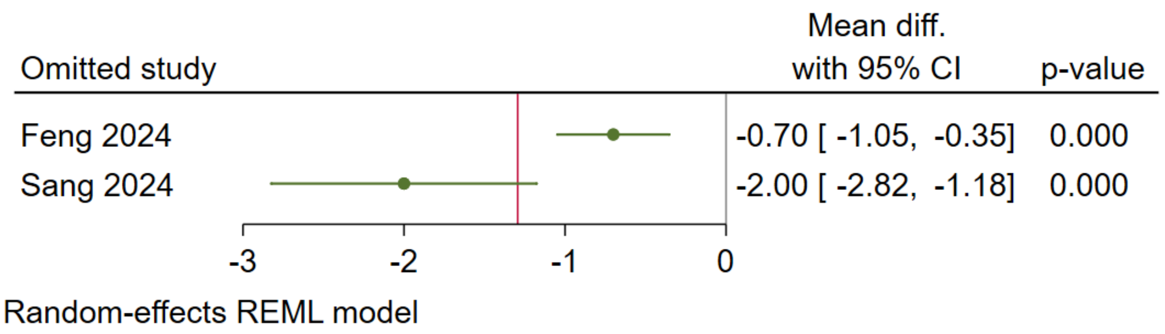

Sensitivity analysis of

Postoperative Drainage Volume

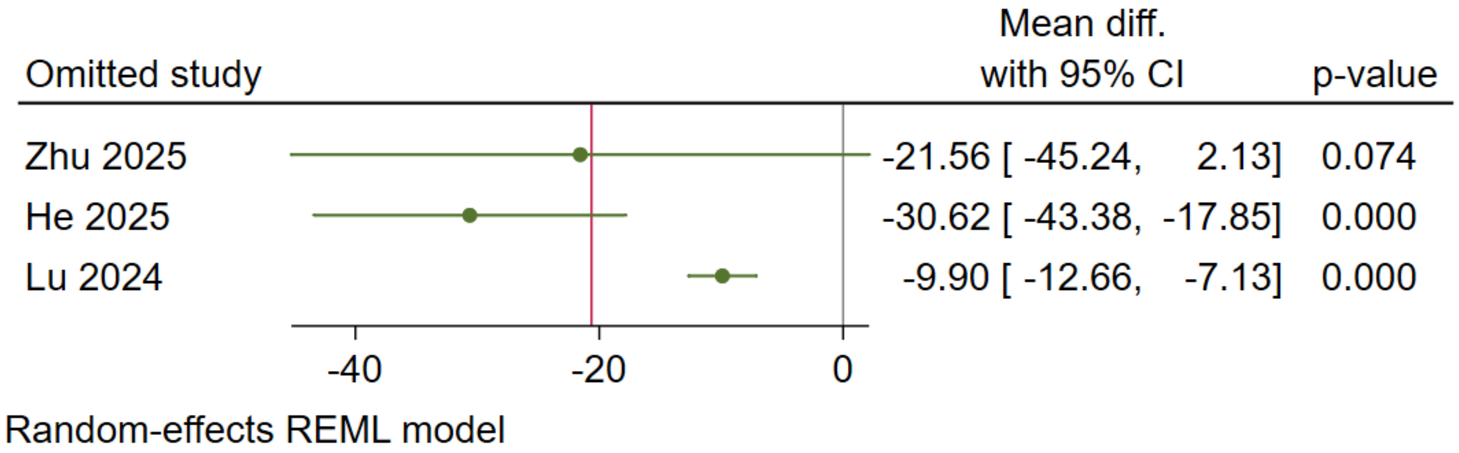

Sensitivity analysis of

Postoperative Ambulation Time

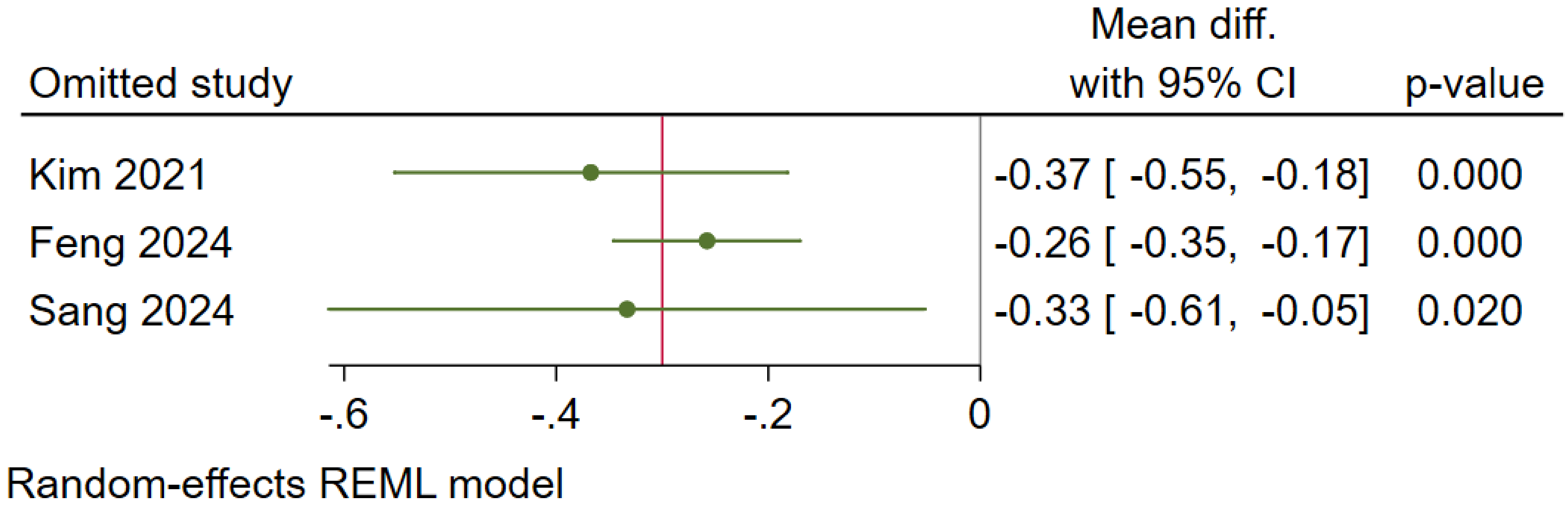

Sensitivity analysis of

Length of Hospital Stay

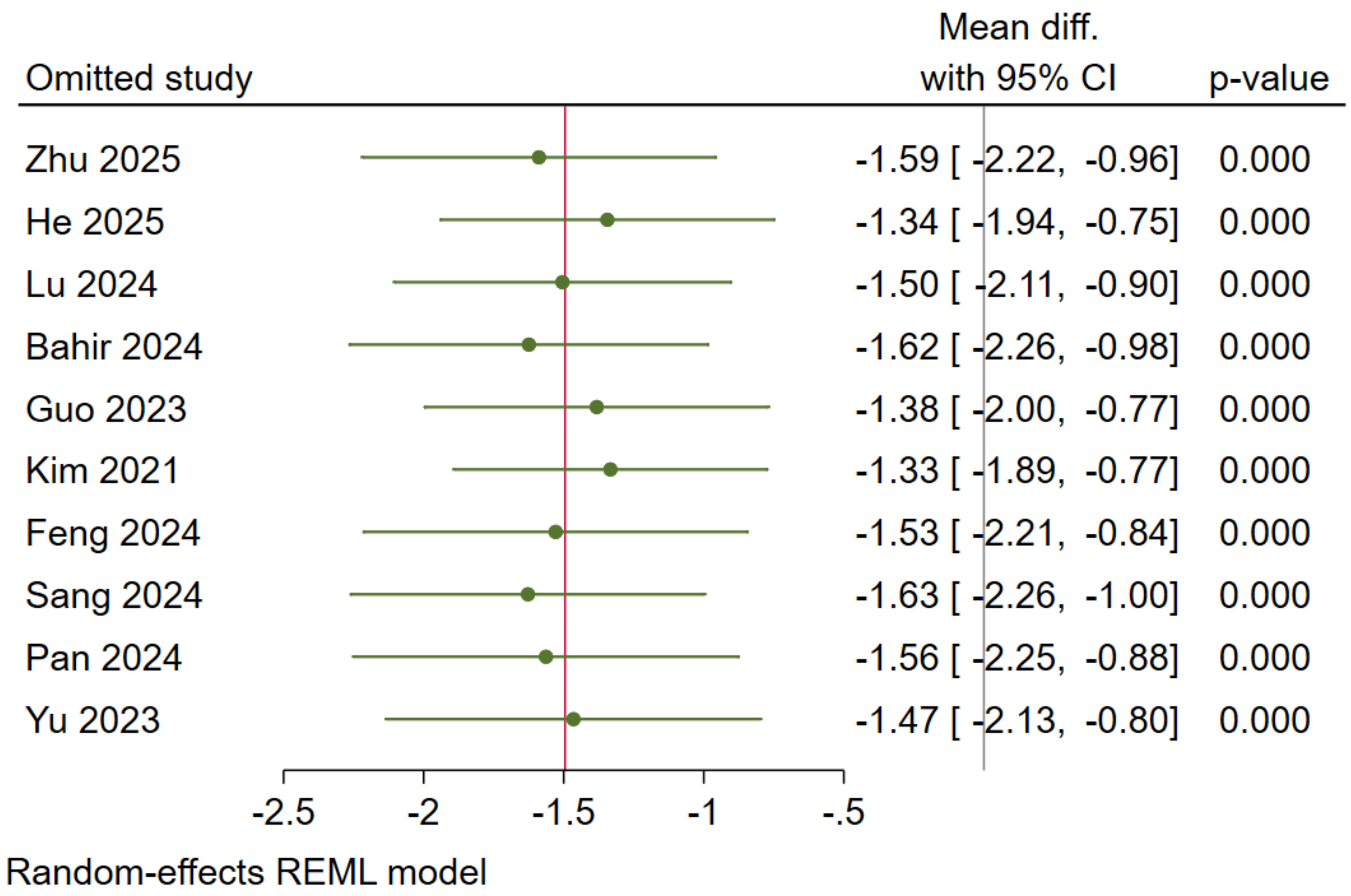

Sensitivity analysis of

Visual Analogue Scale Score for back pain

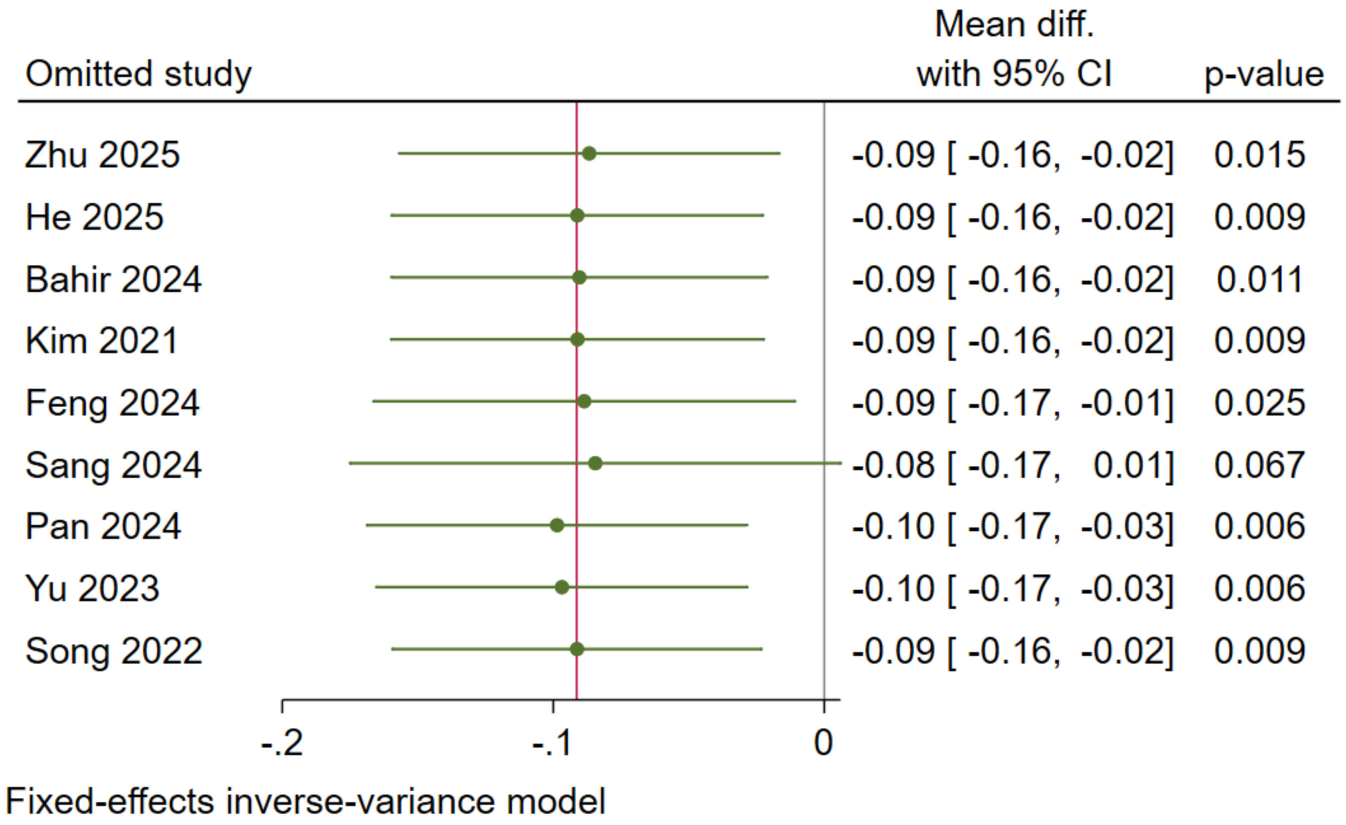

Sensitivity analysis of

Visual Analogue Scale Score for leg pain

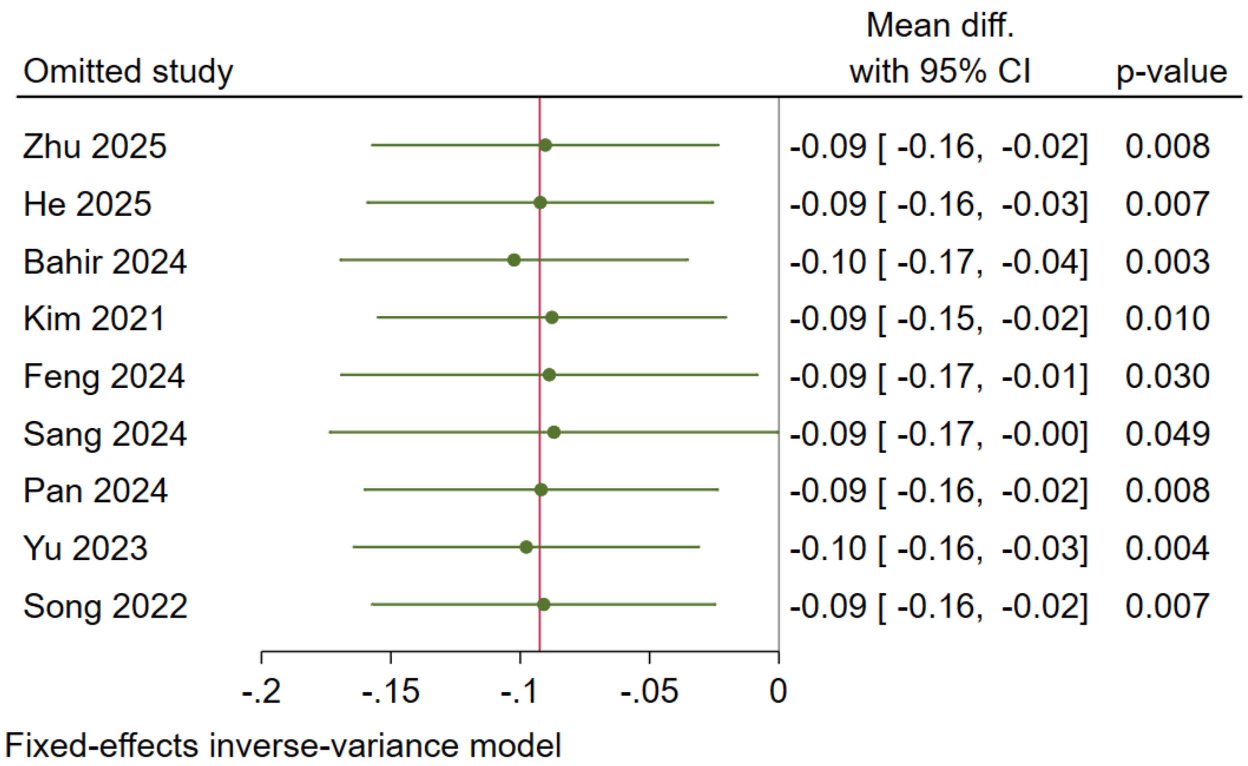

Sensitivity analysis of

Oswestry Disability Index

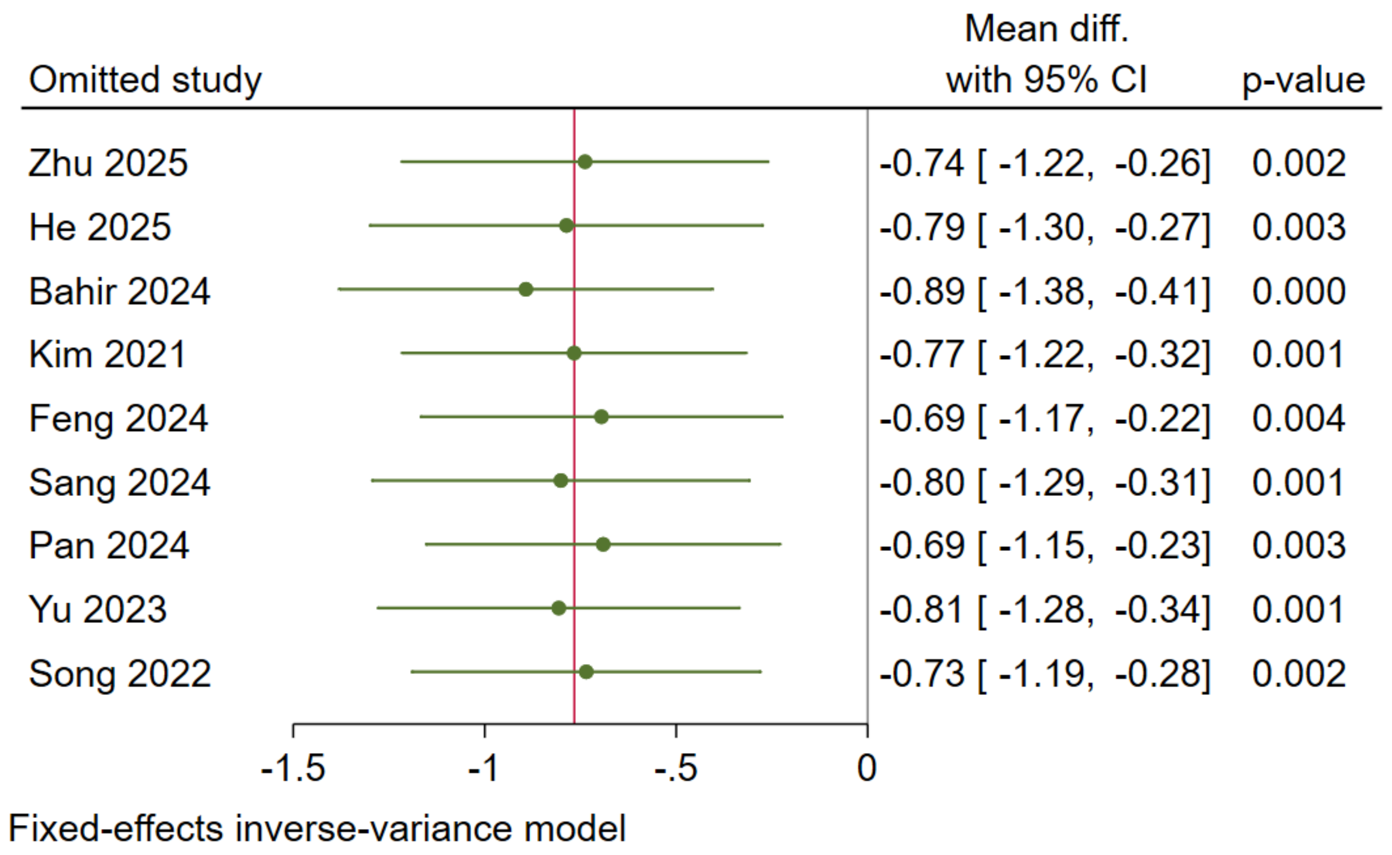

Sensitivity analysis of

Disc Height

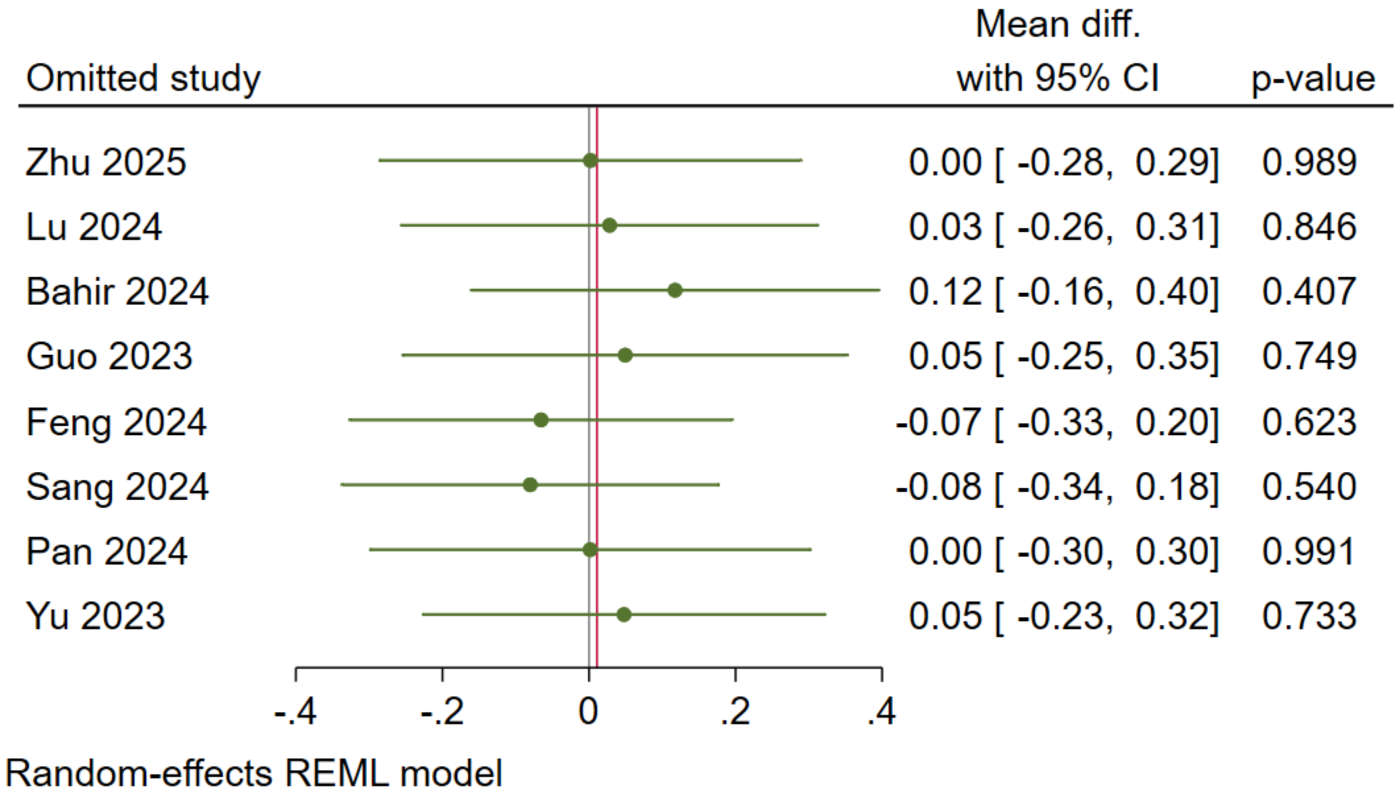

Sensitivity analysis of

Lumbar Lordosis

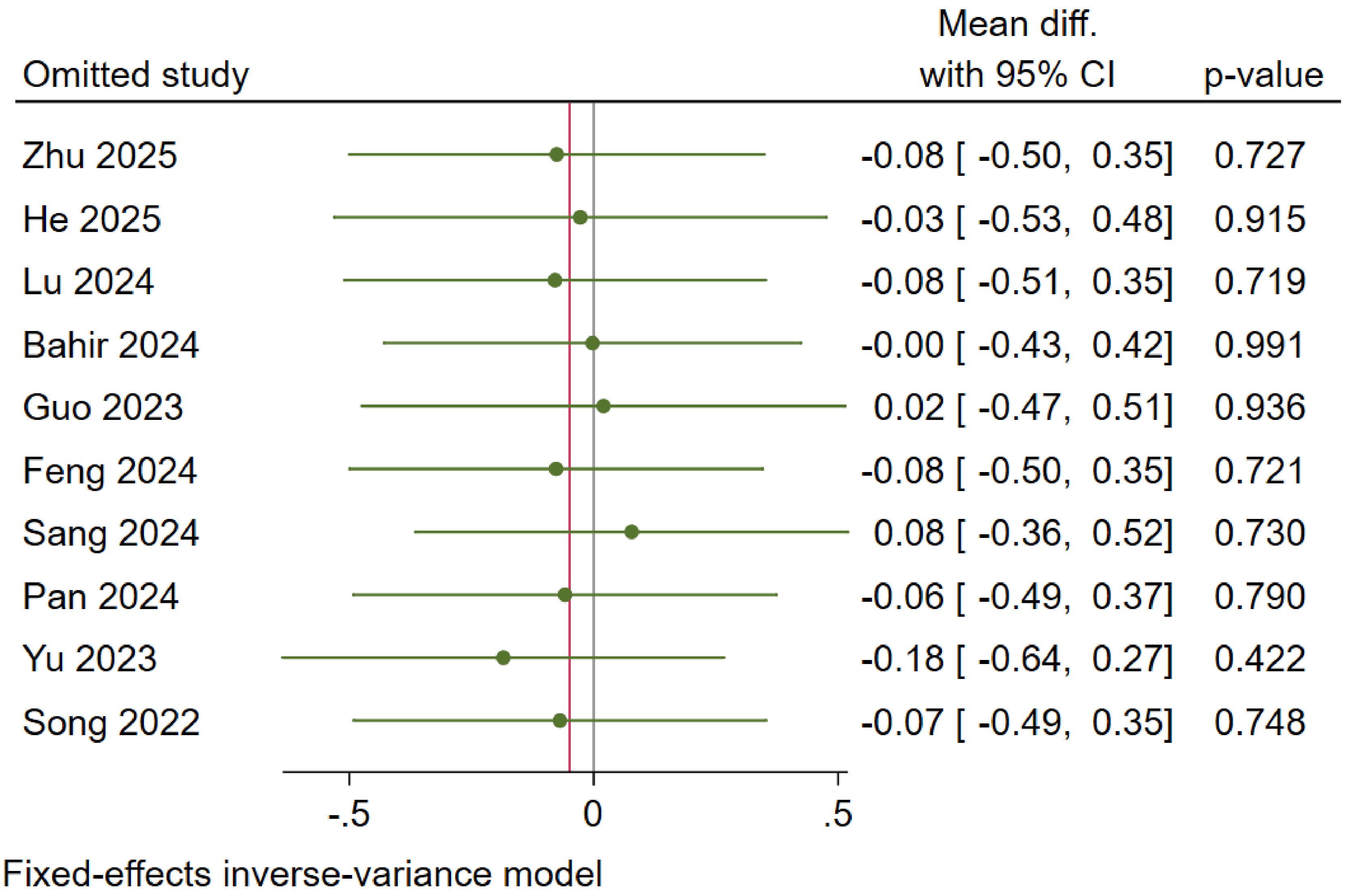

Sensitivity analysis of

Fusion Rate

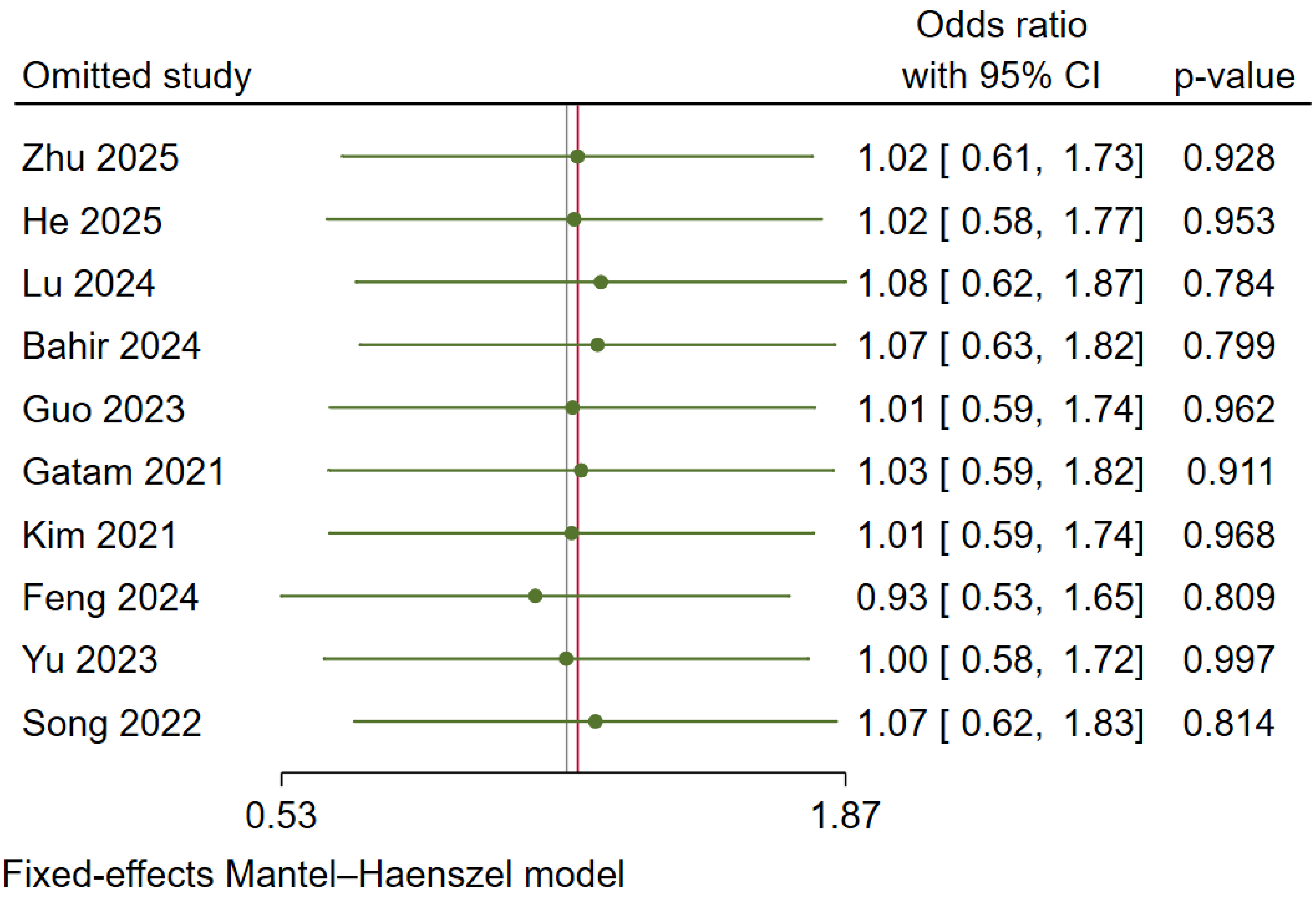

Sensitivity analysis of Complication Rate

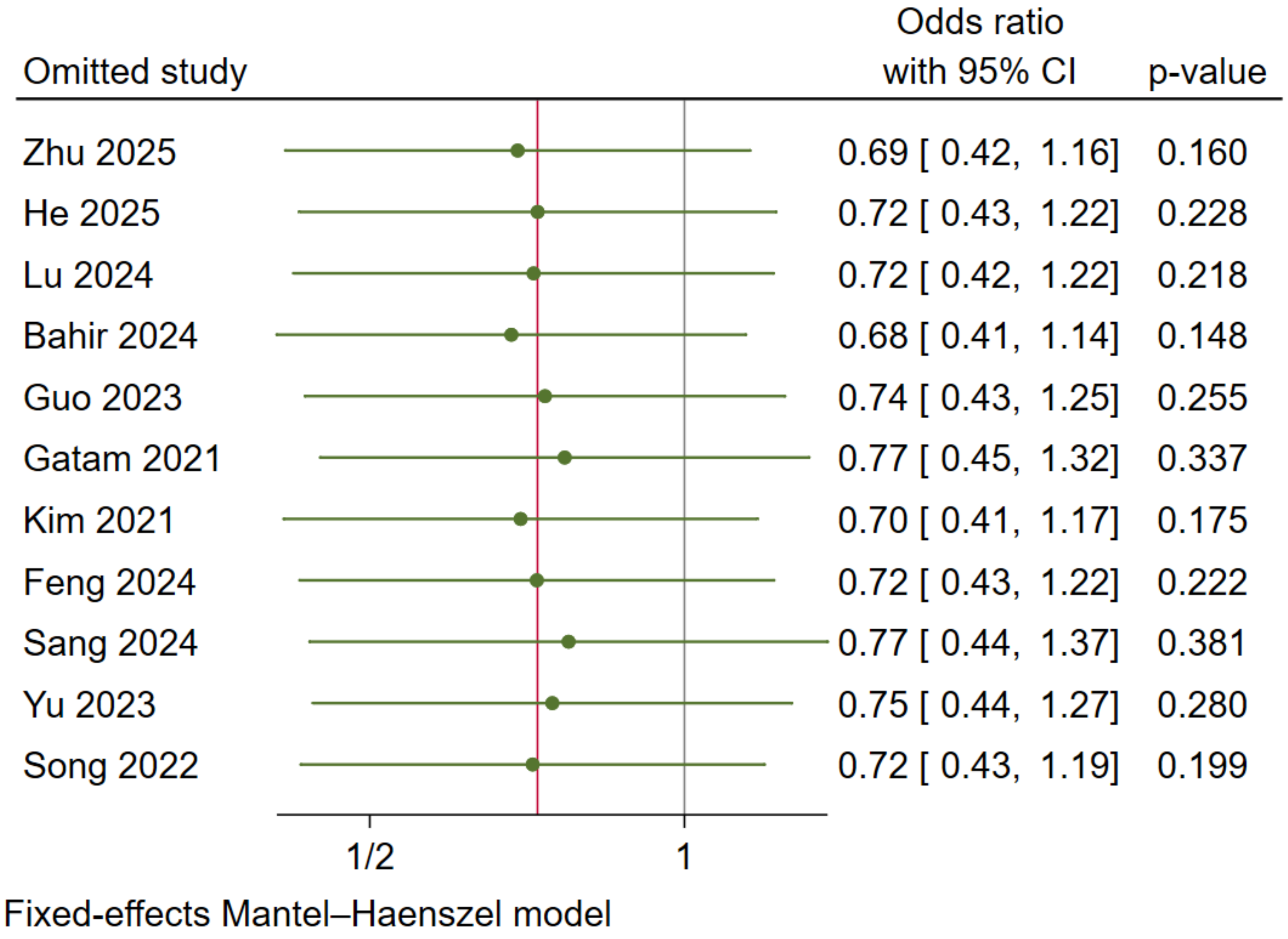

Supplement: Supplementary file 3 [file Data_Sheet_3.pdf]
