# Supplementary material for: Unilateral biportal endoscopic transforaminal lumbar interbody fusion versus minimally invasive transforaminal lumbar interbody fusion for single-level lumbar spondylolisthesis: a systematic review and meta-analysis
Source: Front Med (Lausanne). 2025 Nov 24;12:1686492. doi: 10.3389/fmed.2025.1686492 (PMC12682878; doi:10.3389/fmed.2025.1686492)
Supplement: Supplementary file 4 [file Data_Sheet_4.pdf]

Publication bias of

Duration of Surgery

```
. meta bias, egger

  Effect-size label: Mean diff.
        Effect size: _meta_es
          Std. err.: _meta_se

Regression-based Egger test for small-study effects
Random-effects model
Method: REML

H0: beta1 = 0; no small-study effects
            beta1 =     -0.48
      SE of beta1 =     2.923
                z =     -0.16
       Prob > |z| =    0.8704

.
```

Publication bias of

Intraoperative Blood Loss

```
. meta bias, egger

  Effect-size label: Mean diff.
        Effect size: _meta_es
          Std. err.: _meta_se

Regression-based Egger test for small-study effects
Random-effects model
Method: REML

H0: beta1 = 0; no small-study effects
            beta1 =     -3.00
      SE of beta1 =     3.578
                z =     -0.84
       Prob > |z| =    0.4020

.
```

Publication bias of

Intraoperative Fluoroscopy

```
. meta bias, egger

  Effect-size label: Mean diff.
        Effect size: _meta_es
          Std. err.: _meta_se

Regression-based Egger test for small-study effects
Random-effects model
Method: REML

H0: beta1 = 0; no small-study effects
            beta1 =     -5.35
      SE of beta1 =  7.58e+07
                z =     -0.00
       Prob > |z| =    1.0000

.
```

Publication bias of

Postoperative Drainage Volume

```
. meta bias, egger

  Effect-size label: Mean diff.
        Effect size: _meta_es
          Std. err.: _meta_se

Regression-based Egger test for small-study effects
Random-effects model
Method: REML

H0: beta1 = 0; no small-study effects
            beta1 =     -0.31
      SE of beta1 =     2.329
                z =     -0.13
       Prob > |z| =    0.8944

.
```

Publication bias of

Postoperative Ambulation Time

```
. meta bias, egger

  Effect-size label: Mean diff.
        Effect size: _meta_es
          Std. err.: _meta_se

Regression-based Egger test for small-study effects
Random-effects model
Method: REML

H0: beta1 = 0; no small-study effects
            beta1 =     -3.24
      SE of beta1 =     2.047
                z =     -1.58
       Prob > |z| =    0.1140

.
```

Publication bias of

Length of Hospital Stay

```
. meta bias, egger

  Effect-size label: Mean diff.
        Effect size: _meta_es
          Std. err.: _meta_se

Regression-based Egger test for small-study effects
Random-effects model
Method: REML

H0: beta1 = 0; no small-study effects
            beta1 =     -0.06
      SE of beta1 =     0.942
                z =     -0.06
       Prob > |z| =    0.9504

.
```

Publication bias of

Visual Analogue Scale Score for both back pain

```
. meta bias, egger

  Effect-size label: Mean diff.
        Effect size: _meta_es
          Std. err.: _meta_se

Regression-based Egger test for small-study effects
Fixed-effects model
Method: Inverse-variance

H0: beta1 = 0; no small-study effects
            beta1 =      0.28
      SE of beta1 =     0.661
                z =      0.42
       Prob > |z| =    0.6716

.
```

Publication bias of

Visual Analogue Scale Score for leg pain

```
. meta bias, egger

  Effect-size label: Mean diff.
        Effect size: _meta_es
          Std. err.: _meta_se

Regression-based Egger test for small-study effects
Fixed-effects model
Method: Inverse-variance

H0: beta1 = 0; no small-study effects
            beta1 =      0.24
      SE of beta1 =     0.608
                z =      0.40
       Prob > |z| =    0.6919

.
```

Publication bias of

Oswestry Disability Index

```
. meta bias, egger

  Effect-size label: Mean diff.
        Effect size: _meta_es
          Std. err.: _meta_se

Regression-based Egger test for small-study effects
Fixed-effects model
Method: Inverse-variance

H0: beta1 = 0; no small-study effects
            beta1 =     -0.92
      SE of beta1 =     0.959
                z =     -0.96
       Prob > |z| =    0.3370

.
```

Publication bias of

Disc Height

```
. meta bias, egger

  Effect-size label: Mean diff.
        Effect size: _meta_es
          Std. err.: _meta_se

Regression-based Egger test for small-study effects
Random-effects model
Method: REML

H0: beta1 = 0; no small-study effects
            beta1 =     -0.18
      SE of beta1 =     1.078
                z =     -0.16
       Prob > |z| =    0.8706

.
```

Publication bias of

Lumbar Lordosis

```
. meta bias, egger

  Effect-size label: Mean diff.
        Effect size: _meta_es
          Std. err.: _meta_se

Regression-based Egger test for small-study effects
Fixed-effects model
Method: Inverse-variance

H0: beta1 = 0; no small-study effects
            beta1 =      0.72
      SE of beta1 =     0.644
                z =      1.11
      Prob > |z| =    0.2660

.
```

Publication bias of

Fusion Rate

```
. meta bias, egger
note: declared Mantel–Haenszel method not supported with meta bias; using
      inverse-variance method

  Effect-size label: Log odds-ratio
        Effect size: _meta_es
          Std. err.: _meta_se

Regression-based Egger test for small-study effects
Fixed-effects model
Method: Inverse-variance

H0: beta1 = 0; no small-study effects
            beta1 =     -0.90
      SE of beta1 =     1.458
                z =     -0.62
       Prob > |z| =    0.5357


.
```

Publication bias of

Complication Rate

```
. meta bias, egger
note: declared Mantel-Haenszel method not supported with meta bias; using
      inverse-variance method

  Effect-size label: Log odds-ratio
        Effect size: _meta_es
          Std. err.: _meta_se

Regression-based Egger test for small-study effects
Fixed-effects model
Method: Inverse-variance

H0: beta1 = 0; no small-study effects
            beta1 =      1.38
      SE of beta1 =     1.074
                z =      1.29
       Prob > |z| =    0.1984

.
```
